# Assessment of environmental contamination with soil-transmitted helminths life stages at school compounds, households and open markets in Jimma Town, Ethiopia

**DOI:** 10.1371/journal.pntd.0010307

**Published:** 2022-04-04

**Authors:** Bamlaku Tadege, Zeleke Mekonnen, Daniel Dana, Bizuwarek Sharew, Eden Dereje, Eskindir Loha, Jaco J. Verweij, Stijn Casaert, Johnny Vlaminck, Mio Ayana, Bruno Levecke

**Affiliations:** 1 School of Medicine, Hawassa University, Hawassa, Ethiopia; 2 School of Medical Laboratory Sciences, Jimma University, Jimma, Ethiopia; 3 Department of Translational Physiology, Infectiology and Public Health, Ghent University, Merelbeke, Belgium; 4 Molecular Biology and NTDs Research Center, Jimma University, Jimma, Ethiopia; 5 Chr. Michelsen Institute, Bergen, Norway; 6 Centre for International Health, University of Bergen, Bergen, Norway; 7 Microvida, Laboratory for Medical Microbiology and Immunology, Elisabeth-TweeSteden Hospital, Tilburg, The Netherlands; Imperial College London, UNITED KINGDOM

## Abstract

**Background:**

It remains largely unknown where and how infections with soil-transmitted helminths (STHs; *Ascaris*, *Trichuris*, *Necator* and *Ancylostoma*) occur. We therefore aimed to identify possible sources of infection by assessing the environmental contamination in an STH-endemic area.

**Methods:**

We first performed a series of laboratory experiments designed to optimize a soil straining-flotation method to detect and quantify *Ascaris* and *Trichuris* eggs in soil, and to validate the diagnostic performance of the optimized method when followed by microscopy and qPCR. In a second phase, we applied this method to assess the level of STH contamination in 399 environmental samples collected from 10 school compounds, 50 households and 9 open markets in Jimma Town (Ethiopia). Subsequently, we explored associations between the environmental contamination and both the corresponding STH epidemiology at the level of the schools and the household characteristics. Finally, we assessed the knowledge, attitude and practice (KAP) towards STHs in school children.

**Principal findings:**

Our soil straining-flotation method has an analytical sensitivity of 50 eggs per 100 grams of soil and egg recovery rate of 36.0% (*Ascaris*) and 8.0% (*Trichuris*). The analysis of field samples with both microscopy and qPCR revealed the presence of 8 different helminth species of medical importance, including but not limited to the human STHs. There was a significant association between the environmental contamination and prevalence of any STH infections at the school level only. The KAP indicated a lack of knowledge and awareness of STHs.

**Conclusions/Significance:**

Our optimized straining-flotation method has a moderate diagnostic performance and revealed that life stages of helminths are ubiquitous in the environment, which might be due to the poor sanitary facilities at both the schools and the households, and a poor level of KAP towards STHs. Further research is required to gain more insights into the contribution of these life stages to transmission.

## Author summary

The recent strategies to control the morbidity caused by intestinal worms have mainly been focusing on large scale deworming programs during which drugs are administered to at-risk populations. Although these large-scale deworming programs have been successful in reducing the disease burden, re-infection in the absence of other intervention measures is unavoidable. Although there is a consensus on how infections with intestinal worms are transmitted, it remains unclear where hot spots of infectious life stages can be found in endemic communities, which in turn impedes both the design and the validation of interventions that prevent (re-)infection. We optimized a laboratory method to detect life stages in soil samples and applied this method to examine soil contamination at school compounds, households and open markets in Jimma Town (Ethiopia). Finally, we assessed the household characteristics, and the knowledge, attitude, and practice towards intestinal worms among school children. Generally, our results indicated (i) a moderate performance of our laboratory method, (ii) the ubiquitous presence of life stages in the environment, (iii) a poor level of sanitary facilities at both the schools and the households and (iv) a poor level of knowledge, attitude and practices towards intestinal worms.

## Introduction

Soil-transmitted helminths (STHs) are a diverse group of intestinal worms, including the giant roundworm (*Ascaris lumbricoides*), the whipworm (*Trichuris trichiura*) and the hookworm (*Necator americanus*, *Ancylostoma duodenale* and *Ancylostoma ceylanicum*) [1]. Despite the clear biological differences across these worm species [2]; their transmission is characterized by the same sequence of events: (i) infected individuals excrete worm eggs via their stool into the soil; (ii) under optimal conditions of moisture and temperature the excreted eggs will develop into infectious worm eggs (*A*. *lumbricoides* and *T*. *trichiura*) /larvae (hookworms); (iii) finally infection occurs through oral uptake (eggs) or skin penetration (larvae) of these infectious stages that reside in the soil [3].

Globally, it is estimated that over one fifth of the world population are infected with at least one STH species, resulting in an estimated total disease burden of 3.4 million disability-adjusted life years (DALYs) [4,5]. However, transmission of STHs and the associated disease burden mainly occurs in sub-tropical and tropical countries, where the climate is optimal for development of the infectious stages [6] and where the challenge to maintain an adequate personal hygiene and a lack of sanitation facilitate the transmission. STH-attributable morbidity is mainly associated with moderate-to-heavy intensity infections and disproportionately affects both children and women of child-bearing age [7,8], contributing to impaired growth and cognitive development, malnutrition, anemia, and school absenteeism [9–12]. To fight the STH-attributable disease burden, the World Health Organization (WHO) promotes large-scale deworming programs in which anthelmintic drugs (albendazole and mebendazole) are periodically administered to at-risk populations (e.g., preschool-age children, school-age children, women of childbearing age and workers at higher risk for STH infections, such as tea-pickers and miners), with the ultimate ambition to reduce the prevalence of moderate-to-heavy intensity infections to less than 2% by 2030 [13].

Although these campaigns have been successful in reducing the global STH-attributable morbidity [14,15], re-infection perpetuates in absence of improved water, sanitation and both environmental and personal hygiene, and health education [16–18]. This is in particular when *Ascaris* and *Trichuris* eggs remain viable in the environment for a long period of time [19]. More generally, there is a poor knowledge of the environmental contamination of STH eggs/larvae. Although there is a consensus on how STHs are transmitted, it remains unclear where hot spots of STHs can be found in the communities, which in turn impedes both the design and the validation of water sanitation and hygiene, and health education interventions.

Indeed, studies reporting environmental contamination are mainly focused on animal STHs (e.g., *Toxocara*) with the ultimate goal to assess the risk for zoonotic STH transmission. These studies are conducted in countries where human STHs do not always pose a threat to public health such as Italy [20], Turkey [21], Poland [22] and Portugal [23]. Only a few studies aimed to gain insights into potential hot spots for human STHs in endemic settings, such as Cameroon [24], Kenya [25], Brazil [26], Nepal [27], Indonesia [28] and Thailand [29]. In these studies, samples were taken around households [25,29], playgrounds in school compounds [24,30], latrines [24,31] and markets [30], each of the studies highlighting the presence of STHs eggs/larvae (*Ascaris*: up to 27% [27], *Trichuris*: up to 77.0% [29], and hookworms: up to 27.5% of the samples [30]). Moreover, across these studies a plethora of methodologies have been applied for both collecting and analyzing soil samples, which makes it difficult to compare study results. For example, different amounts of soil (30–500 grams [27,28,32–37]) and soil types (clay *vs*. sandy and loam [38–43]) were collected at different depths (0–10 cm [32–34,37]) and at different points in time (dry *vs*. rainy season [28,29,33,34,42]). In general, the detection and quantification of STH eggs/larvae in soil are based on a series of steps that allow separating the STH eggs/larvae from soil particles, such as straining of soil and flotation of eggs/larvae. Yet, important differences across the currently applied methods can be noted in terms of pre-treatment (adding of surfactant [25,44] or NaOH [45]), mesh size (50–250 μm; [21,33,35,46]), type and density flotation solution (FS; NaCl [33], ZnSO_4_ [30], sucrose [46], NaNO_3_ [47] and MgSO_4_ [48]), centrifugal force at which flotation solution is centrifuged (1,000–2,500 rpm [30,49,50]) and time allowed for eggs to float (5–30 min [37,44,47,51,52]). Once purified, eggs/larvae are mostly detected and quantified by means of microscopic examination. Only in rare cases, DNA is extracted and subjected to STH species-specific nucleic acid amplification tests (e.g., PCR [53–55] and qPCR [56,57]). These DNA-based assays are not only considered to be more sensitive, they also allow for differentiation of the different animal and human hookworm species [55]. On top of these clear differences in methodologies, the diagnostic performance of the entire sample analysis process is mostly unknown [58], which further complicates the interpretation of the results.

In the present study, we first performed a series of laboratory experiments designed to optimize an in-house soil straining-flotation method to detect and quantify *Ascaris* and *Trichuris* eggs in soil and to validate the diagnostic performance of the optimized method when followed by both microscopy and qPCR. In a second phase, we applied this method to assess the level of STH contamination in environmental samples collected from school compounds, households and open markets in Jimma Town (Ethiopia), an area that is endemic to STHs [59,60]. Subsequently, we explored associations between the environmental contamination and both the corresponding STH epidemiology at the level of the schools and the household characteristics. Finally, we assessed the knowledge, attitude and practice (KAP) towards STH among school children.

## Methods

### Ethical statement

The protocols for the assessment of the environmental STH contamination, STH infections in children, household characteristics and KAP towards STH infections were approved by the Institutional Review Board of Jimma University, Ethiopia (reference numbers IHRPGD/680 and IHRPGD/466/2020). The school authorities, teachers, parents and the children were informed about the purpose and procedures of the study based on the informed consent, and adequate time was given to make an informed consent. A written informed consent was obtained from the parents/guardians. An additional separate written consent was secured from children older than 12 years. Consent forms were prepared in English, translated into the two commonly used local languages (Afaan Oromo and Amharic) and a copy was handed over to the children’s parents/guardians. Only those children who were willing to participate and whose parents or guardians signed the written informed consent form were included in the study. In case of Illiteracy, a thumb print was considered as a signature.

### Optimization of an in-house soil straining-flotation method

**Fig 1:**
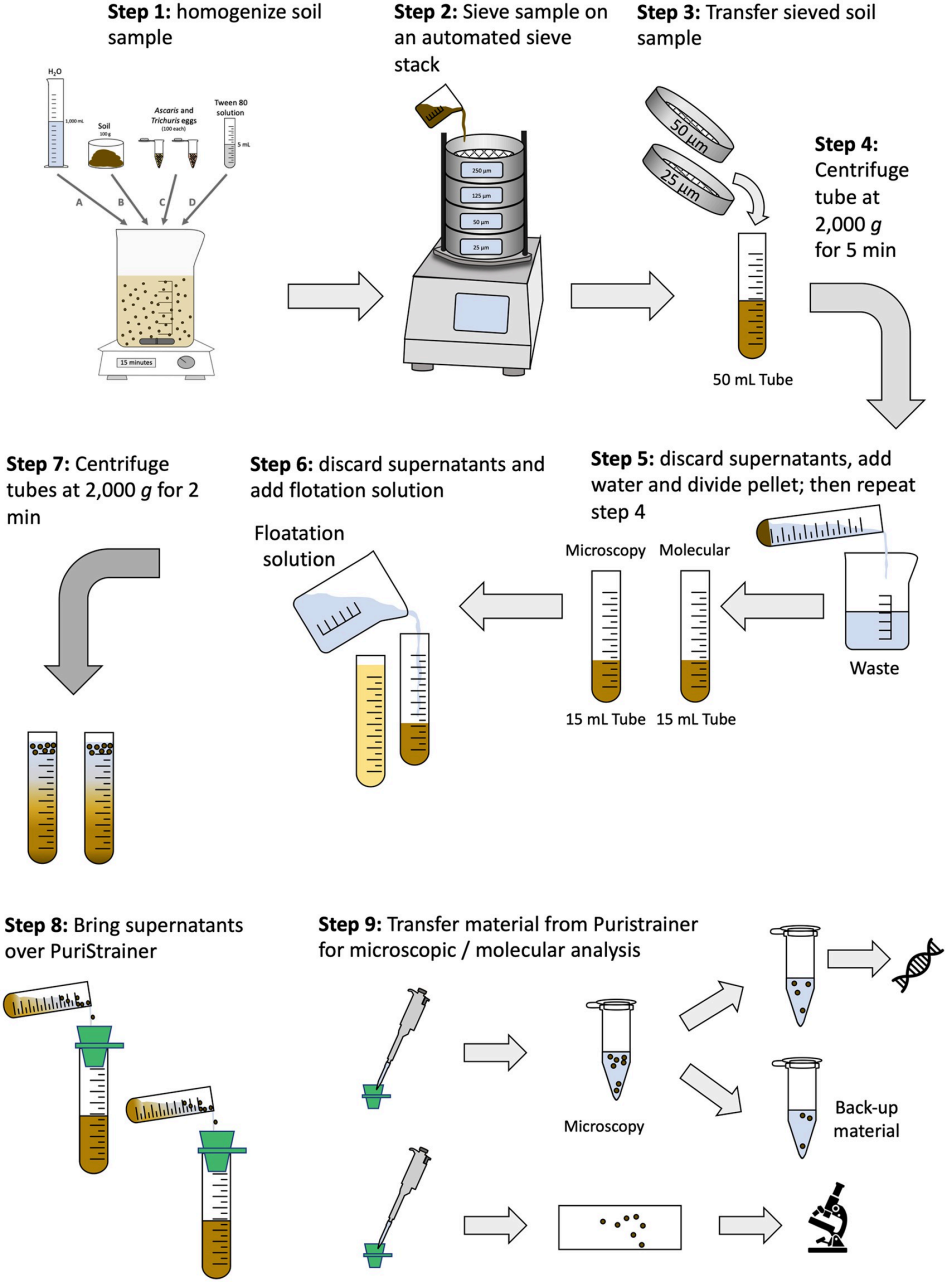
The different steps of the optimized straining-flotation method to detect and quantify STH eggs/larvae in soil. The optimized straining-flotation method consists of 9 consecutive steps, including the homogenization of the soil sample (**step 1**), sieving of the sample (**step 2**), the transfer of the sieved soil into a 50 mL tube (**step 3**), centrifugation of this test tube at 2,000 *g* for 5 min (**step 4**), discard supernatant, add water to bring the pellet to suspension, equally divide suspension over two 15 mL tubes, and repeat the centrifugation step (**step 5**), discard supernatants and add flotation solution to each of the test tubes (**step 6**), centrifuge the tubes at 2,000 *g* for 2 min (**step 7**) and put the supernatants over a PuriStrainer sieve (**step 8**), transfer the material from the PuriStrainer sieve to a microscopic slide or in a 2 mL tube for microscopic or molecular analysis, respectively (**step 9**).

In the present study, we opted to optimize and validate our own soil straining-flotation method, based on the general laboratory procedures that are known to enhance both the purification and the concentration of STH life stages from environmental samples. Fig 1 provides a schematic overview of the different consecutive steps to detect and quantify STH eggs in soil samples applying our in-house optimized straining-flotation method. To optimize this method, we separately conducted 5 experiments during which we measured the egg recovery rate by microscopy, each building on the results from the previous experiment. We first determined the FS and the period of floating that maximized the recovery of *Ascaris* and *Trichuris* eggs (*flotation experiment*). Subsequently, we verified whether we could further improve the egg recovery rate by varying the centrifugal force (*centrifugal force experiment*) and adding detergents (*Tween experiment*), or whether the amount of sieved soil had an impact on the egg recovery rate (*volume soil experiment*). Finally, we verified whether we could further simplify the recovery of eggs from the flotation test tube without affecting the egg recovery rate (*strainer experiment*). For each of these spiking experiments we used cultured porcine *Ascaris suum* and *Trichuris suis* eggs. These eggs were collected from adult *A*. *suum* and *T*. *suis*, and were cultured in 2% K_2_Cr_2_O_7_ at room temperature for at least 1.5 months to develop into infectious stages. The cultures were stored at room temperature at the Laboratory of Parasitology (Ghent University). Prior use they were neutralized by incubating them at 60°C for 1 h. The egg concentration of the two cultures were determined by examining 10 times 20 μL. The soil used in the experiments originated from Jimma Town and was sterilized at 150°C for 30 min.

#### Flotation experiment

A total of 24 test tubes of 15 mL was filled with different FSs, including eight tubes with 10 mL NaCl (specific gravity = 1.2), eight tubes with 10 mL MgSO_4_ (specific gravity, 1.28) and eight tubes with 10 mL ZnSO_4_ (specific gravity = 1.35). The number of tubes (replicates) was not based on a formal sample size calculation, rather it was based on pragmatic considerations. A total of 200 cultured eggs of porcine *A*. *suum* (100 eggs) and *T*. *suis* (100 eggs) were added to each test tube, after which the tubes were vortexed and subsequently centrifuged at 2,000 *g* for two min. Subsequently, more FS was added to form a meniscus and a cover slip was placed on top of the meniscus allowing eggs to float. To assess the impact of the period at which those eggs are allowed to float in the test tube, we removed the cover slip at different time points (5, 10, 15 and 30 min). For each time point, we performed six replicates (two replicates for each FS). In addition, we assessed the impact of serial flotation. To this end, we placed a second cover slip after removing the first and a third cover slip after removing the second. Before placing the next cover slip, we added FS to form a new meniscus. Each of these 2 additional cover slips was left for 15 min. All cover slips were placed on a microscopic slide and were examined using a compound microscope (100x magnifications). The number of *Ascaris* and *Trichuris* eggs per cover slide were recorded, resulting in a total of 72 (= 3 FSs x 8 tubes x 3 cover slips) egg counts per STH species.

#### Centrifugal force experiment

To assess the impact of centrifugal force on egg recovery, we determined the recovery rate of eggs when flotation test tubes were centrifuged at 1,000 and 2,000 *g*. To this end, 16 test tubes were filled with 10 mL of ZnSO_4_ (the optimal FS derived from the flotation experiment). A total of 100 porcine *Ascaris* and 100 *Trichuris* eggs were added to ZnSO_4_, after which the test tubes were centrifuged for two min at 1,000 *g* (eight tubes) or 2,000 *g* (eight tubes). Subsequently, more ZnSO_4_ was added to form a meniscus and a first cover slip was placed on the meniscus allowing eggs to float. After 30 min, this cover slip was immediately placed on a microscopic slide. A second and a third cover slip were placed and removed with a 15 min interval (the optimal examination strategy derived from the flotation experiment). All cover slips were placed on a microscopic slide and were examined using a compound microscope. The number of *Ascaris* and *Trichuris* eggs per cover slide were recorded, resulting in a total of 48 (= 1 FS x 2 x centrifugal forces x 8 tubes x 3 cover slips) egg counts per STH species.

#### Concentration of Tween experiment

To assess the impact of detergent on egg recovery from soil we determined the recovery of eggs when the ZnSO_4_ did include detergent. To this end, 16 test tubes were filled with 10 mL of ZnSO_4_, with (eight tubes) or without (eight tubes) Tween 80 (0.05%). A total of 100 *Ascaris* and 100 *Trichuris* eggs were added to the ZnSO_4_ and mixed, after which the test tubes were centrifuged for two min at 2,000 *g* (the optimal examination strategy derived from the centrifugal force experiment). Subsequently, more ZnSO_4_ was added to form a meniscus and a first cover slip was placed on the meniscus allowing eggs to float. As in the centrifugal experiment, three cover slips were examined per tube, resulting in a total of 48 egg counts (2 concentrations of detergent x 1 FS x 1 x centrifugal force x 8 tubes x 3 cover slips).

#### Volume of soil experiment

So far, we did not add any soil to the tube. To assess the effect of volume of soil on egg recovery, we evaluated the egg recovery rate from one mL and three mL of strained soil. The volume rather than the mass of soil was preferred as it was expected that adding a volume would impact the density of the ZnSO_4_. The choice of three mL as the highest possible volume was based on three aspects, namely 100 grams of soil corresponded with an end volume of six mL, further processing this volume would result in a reduction in the specific gravity of the ZnSO_4_ (from 1.35 to 1.21; for three mL this is 1.28) and the need for splitting the processed soil sample into two subsamples (one for microscopic examination and one for qPCR). A total of 16 test tubes were filled with 10 mL of ZnSO_4_ and with one mL (12 tubes) or three mL of sterilized soil (12 tubes). A total of 100 porcine *Ascaris* and 100 *Trichuris* eggs were added to the ZnSO_4_ and mixed, after which the test tubes were centrifuged for two min at 2,000 *g*. Subsequently, more ZnSO_4_ was added to form a meniscus and a first cover slip was placed on the meniscus allowing eggs to float. Similarly, like the previous experiments, three cover slips were examined per tube (1^st^ coverslip at 30 min; 2^nd^ coverslip slide at 15 min; and 3^rd^ coverslip at 15 min), resulting in a total of 72 egg counts (2 volume of soil x 1 FS x 1 x centrifugal force x 12 tubes x 3 cover slips).

#### Strainer experiment

From the flotation experiment it was noticed that a serial flotation is required to maximize the egg recovery rate. Because this became a time-consuming process, we verified whether we obtained similar recovery rates when the content (ZnSO_4_ and eggs) of the centrifuged test tubes is strained over a small sieve, after which the filtrate is transferred to a microscopic slide. Applying this strategy would remove the repeated examination of cover slips over the course of one hour (1^st^ coverslip removed after 30 min, 2^nd^ coverslip at 45 min = (30 min + 15 min) and 3^rd^ at one hour (30 min + 15 min +15 min)). To this end, 16 test tubes were filled with 10 mL of ZnSO_4_ and a total of 100 *Ascaris* and 100 *Trichuris* eggs were added and mixed. All test tubes were centrifuged for two min at 2,000 *g*. Half of the tubes were processed as described in the previous experiments (three cover slips at 30 min, 15 min and 15 min intervals). The content of the other half of the tubes was strained through a pluriStrainer (mesh size 20 μm). The sieve was washed with tap water, after which the filtrate was aspirated using a micropipette and transferred to a microscopic slide. Finally, four cover slips were placed on slide (this was required to cover the entire aspirate), and the number of *Ascaris* and *Trichuris* were counted.

#### Diagnostic performance of the optimized straining-flotation method

The diagnostic performance (ability to detect at least one egg and to estimate the concentration/contamination of eggs in 100 grams of soil) of the optimized method was evaluated over a wide range of egg concentrations (10, 25, 50 and 100 eggs per 100 grams of soil). A total of 100 grams of sterilized soil were mixed with one L tap water and five mL of 0.05% Tween 80. Then, 100 porcine *Ascaris eggs* and 100 *Trichuris* eggs were added. The suspension was further homogenized for 15 min using a magnetic stirrer plate at 50 rpm. Subsequently, the suspension was strained over a tower of four sieves with different mesh sizes (250 μm, 125 μm, 50 μm and 25 μm) ordered from top to bottom (largest mesh sizes at the top). These sieves were incorporated into the Vibratory Sieve Shaker of the Retsch GmbH machine, and were washed and shaken for 20 min at an amplitude of 0.56 mm. The filtrate on the 50 μm and 25 μm sieves was washed and transferred to a 50 mL test tube. These tubes were then centrifuged for 5 min at 2,000 *g*. Then the supernatant was removed, and the sedimented soil was equally divided into two 15 mL test tubes. Both test tubes were processed using ZnSO_4_ as FS without Tween 80, applying a centrifugal force of 2,000 *g* for two min and using the pluriStrainer (mesh size 20 μm) to simplify the egg count process. The eggs from one tube were microscopically counted, while the eggs from the other tube were preserved at– 20°C and shipped to the Laboratory of Parasitology (Faculty of Veterinary Medicine, Ghent University, Belgium) where the samples DNA-extraction was initiated. At the Microvida of the Laboratory for Medical Microbiology and Immunology, Elisabeth-TweeSteden Hospital, Tilburg (The Netherlands), DNA extraction was completed, and the detection of *Ascaris* and *Trichuris* DNA applying qPCR was performed as previously described [61–63]. For each egg concentration, this process was repeated eight times. Only half of material collected from 20 μm strainers (**step 8** in Fig 1) was processed with qPCR. Processing half of the material was a strategic choice to have a back-up if the sample process failed.

### Application of the optimized straining-flotation method on field samples

In total, three surveys were performed to assess the environmental contamination of STH eggs/larvae in Jimma Town (Southwest Ethiopia), including one involving 10 school compounds, one including 50 households and one conducted at nine open markets. Previous studies highlight that STHs are highly prevalent in Jimma Town, with the prevalence in schools of any STH ranging from 8.3% to 55.0% [59,60]. In the following paragraphs we will discuss each of the surveys in more detail.

#### School compounds

A total of 10 governmental schools in Jimma Town were included in this survey. The selection of these schools was based on their involvement in previous STH epidemiological surveys [59,60] and drug efficacy trials [59,64]. At each school, soil samples were collected from the playgrounds, around the latrine and in the classrooms. From playgrounds, we selected the six most used playground sites and collected 6 soil samples (each 300 grams). Each sample was transferred into a polyethylene bag and labeled with both the school name and the sample collection site. Around the latrines, we collected five soil samples (each 300 grams), one from the entrance for boys, one from the entrance for girls, one in front of the latrine, one behind the latrine and one from the backyard of the latrine. Finally, six classrooms (one classroom per each of the six grades) were identified. At each classroom the entire space was swept, and 300 grams of dust/soil were transferred into a polyethylene bag. Based on this, a total of 170 soil samples (= 10 schools x (6 playground samples + 5 latrine area samples + 6 classroom samples) was collected.

#### Household

The environmental contamination of STH eggs/larvae was assessed at 50 households. These households represent the house of 50 school children that were randomly selected based on their infection status (27 children not excreting STH eggs and 23 children excreting STH eggs based on a single Kato-Katz thick smear) from five out of the 10 aforementioned schools. The five schools were selected based on (i) the distance between schools (only one school per kebele (the smallest unit of government administration) was included), (ii) the social-economic status of the communities they serve (schools serving both rural and urban communities were excluded), and (iii) their availability to participate in the study (schools where students had to take exams during the sample collection were excluded). The screening of the school children was part of a larger study designed to assess the impact of mass drug administration by both stool microscopy (Kato-Katz thick smear) and serology [60]. Four sites at the house of the selected school children were sampled, including the entrance of the house and latrine, the kitchen and the backyard, resulting in a total of 200 samples (50 households x 4 samples).

#### Open market

The mothers of the 50 randomly selected children were interviewed to determine where they buy their vegetables, fruits and cereals. Based on these interviews nine open markets were identified. At each open market, three soil samples were collected from each area where products were presented to customers (vegetables, fruits and cereals). In one big open market, 6 soil samples were collected, resulting in 30 soil samples (= 8 markets x 3 samples + 1 market x 6 samples).

#### Collection of soil samples

We used a shovel to collect the top layer of soil (0–3 cm). We limited the collection of soil to the superficial soil only; as we assumed that children would interact with these layers only and as previous studies have also shown that the infectious *Ascaris* and *Trichuris* eggs are not commonly found beyond this depth [65,66]. At each site, approximately 300 grams of the topsoil was collected from areas free of grass. Samples were collected during both the dry and wet season, and hence the ratio water to soil will be different over seasons (it is expected that 300 grams of soil in the wet season includes more water). To avoid contamination across sample sites, the shovel was cleaned between each sampling sites with a broom to remove the dust. In the case of classrooms, the whole classroom was first swept with a broom, after which the dust from the entire classroom was collected. All the samples were kept in a polyethylene bag that was clearly labeled with date, place and site of collection. All samples were collected in a box and transported to the Neglected Tropical Disease Laboratory of Jimma University.

#### Examination of the soil samples

Prior to the sample processing, we put all samples overnight at room temperature. Subsequently, samples were homogenized to ensure a more uniform distribution of eggs/larvae. Finally, a subsample of 100 grams soil was processed. We processed all the samples applying the optimized and validated straining-flotation method (see Fig 1). Briefly, 100 grams of soil were mixed with one L tap water and five mL of 0.5% Tween 80. The suspension was further homogenized for 15 min on a magnetic stirrer plate at 50 rpm. Then, the suspension was strained over a tower of four sieves with different mesh sizes (250 μm, 125 μm 50 μm and 25 μm) ordered from top to bottom (sieves with largest mesh size on top). These sieves were incorporated into the Vibratory Sieve Shaker of the Retsch GmbH machine and were washed and shaken at an amplitude of 0.56 mm for 20 min. The filtrate on the 50 μm and 25 μm was washed and transferred to a 50 mL test tube. These tubes were then centrifuged for five min at 2,000 *g*. Then the supernatant was removed, and the remaining soil was equally divided and transferred into two 15 mL test tubes labeled one for microscopy and the other for molecular analysis. Both test tubes were processed using ZnSO_4_ as FS without Tween 80, applying a centrifugal force of 2,000 *g* for two min. Then, the supernatants of the tubes were strained separately over a pluriStrainer (mesh size 20 μm). The sieves were washed with tap water, after which the filtrate was aspirated using a micropipette. For the first tube, the filtrate was transferred to a microscopic slide and the number of *Ascaris*, *Trichuris* and hookworm-like eggs/larvae were counted. For the second tube, the supernatant was transferred into a two mL tube, which was preserved at -20°C and shipped to the Laboratory of Parasitology (Faculty of Veterinary Medicine, Ghent University, Belgium). At the Laboratory of Parasitology, the samples were prepared for DNA-extraction. At the Microvida the Laboratory for Medical Microbiology and Immunology, Elisabeth-Twee Steden Hospital, Tilburg (The Netherlands), a total of 432 DNA extraction was completed and the detection of DNA of the different STHs (differentiating *Necator* from *Ancylostoma*), *Strongyloides*, *Taenia* (differentiating *T*. *saginata* and *T*. *solium*), *Enterobius vermicularis*, and *Hymenolepis* nana applying qPCR were performed. The protocols for qPCR assays are described elsewhere [61–63].

#### Questionnaires

Two separate questionnaires were developed, one to obtain information on the characteristics of the households, and one on the KAP towards STH in school children. To obtain information on the household characteristics, we interviewed the mother of the household. In case the mother was not present, we interviewed the father or another head of the household. Through this questionnaire we obtained information on the age of the youngest child, presence of water at home, presence of animals in the compound, both presence and type of latrine (presence of walls, door and roof), the hygienic situation of the latrine (water in latrine, flies observed in/around latrine, visible excreta observed on latrine floor) and the defecation habits of the different family members beyond the latrine. For the KAP, we interviewed 422 school children (age 12 to 18 years old) across five out of the 10 schools. The complete questionnaires can be found in S1 Info (household characteristics) and S2 Info (KAP).

### Statistical data analysis

#### Optimization of the straining-flotation method flotation procedures

For each experiment, we calculated the median egg recovery rate (= proportion (in %) of the 100 *Ascaris* and 100 *Trichuris* eggs recovered) across the different variables. In addition, any significant differences in egg recovery rate were assessed. Given the small sample size we used the non-parametric tests (Wilcoxon rank sum test) only. In case of multiple pair-wise comparisons, the Bonferroni correction was applied on the level of significance. In absence of a clear significant difference in egg recovery rate, either the cheapest or least labor-intensive procedure was incorporated into the final procedure. In case there was no obvious difference in cost/labor, the procedure with the highest egg recovery rate was incorporated.

#### Diagnostic performance of the optimized straining-flotation method

We calculated the sensitivity (= proportion (in %) of the samples correctly classified as positive) for each of the levels of egg contamination (10 eggs / 100 grams of soil, 25 eggs / 100 grams of soil, 50 eggs / 100 grams of soil and 100 eggs / 100 grams of soil) and for the two diagnostic methods (microscopy and qPCR), separately. To explore the performance of the optimized straining-flotation method and to quantify the level of egg contamination, we determined the egg recovery rate across the different levels of egg contamination.

#### Application of the optimized straining-flotation method on field samples

The proportion of contaminated samples and the median level of egg/larvae contamination was determined separately for each cluster (school compounds, household, open market) and sample location (school compounds: playground, latrine, and classrooms; households: entrance of the house, entrance latrine, kitchen, and backyard; markets: vegetables, fruits and cereals). A sample was considered contaminated when either microscopy or qPCR revealed the presence of STH eggs or DNA.

To explore associations between the environmental contamination and STH infections, we first determined the correlation between the environmental contamination and the corresponding STH epidemiology at the level of the schools. To this end, we determined the Spearman correlation coefficient between the proportion of the soil samples containing STH eggs/DNA and the prevalence of STH at the school level. The data on the STH epidemiology was obtained through a study that was designed to compare both microscopy and two in-house serological assays [60], and that was completed 4–8 months prior to the assessment of the environmental contamination (October–December 2018 *vs*. March–June 2019).

Finally, we compared the test results between microscopy and qPCR. In absence of a gold standard (method that has a sensitivity and specificity of 100%), we considered the composite reference standard (CRS; [67,68]) as a proxy for a gold standard to calculate the sensitivity of the different diagnostic methods. This CRS method classified a sample as positive if eggs or DNA are found at least with one of the diagnostic methods and as negative if no eggs or DNA are found with all methods. The sensitivity was determined for all human helminths detected in soil samples assuming a 100% specificity for each method, as indicated by the morphology of the eggs or by the species-specific primers/probes used in the qPCR assays, except for hookworm and *Strongyloides*. For these helminths, the detection is only based qPCR (it is difficult to differentiate them from other free-living/plant parasitic worms based on their morphology only).

#### Questionnaires

For both questionnaires (household characteristics and KAP), we summarized the results applying standard descriptive statistics (proportions for categorical variables; median and range for continues/integer variables). We assessed whether samples of households where infected children lived were more likely to contain STH eggs/larvae. For this, we using a Chi^2^-test to assess difference in the presence of STH at the household level and the Wilcoxon rank sum test (two level variables) /Kruskall Wallis (variables with more than two levels) for the difference in mean number of STH eggs at the household level.

## Results

### Optimization of the in-house straining-flotation method procedures

**Fig 2:**
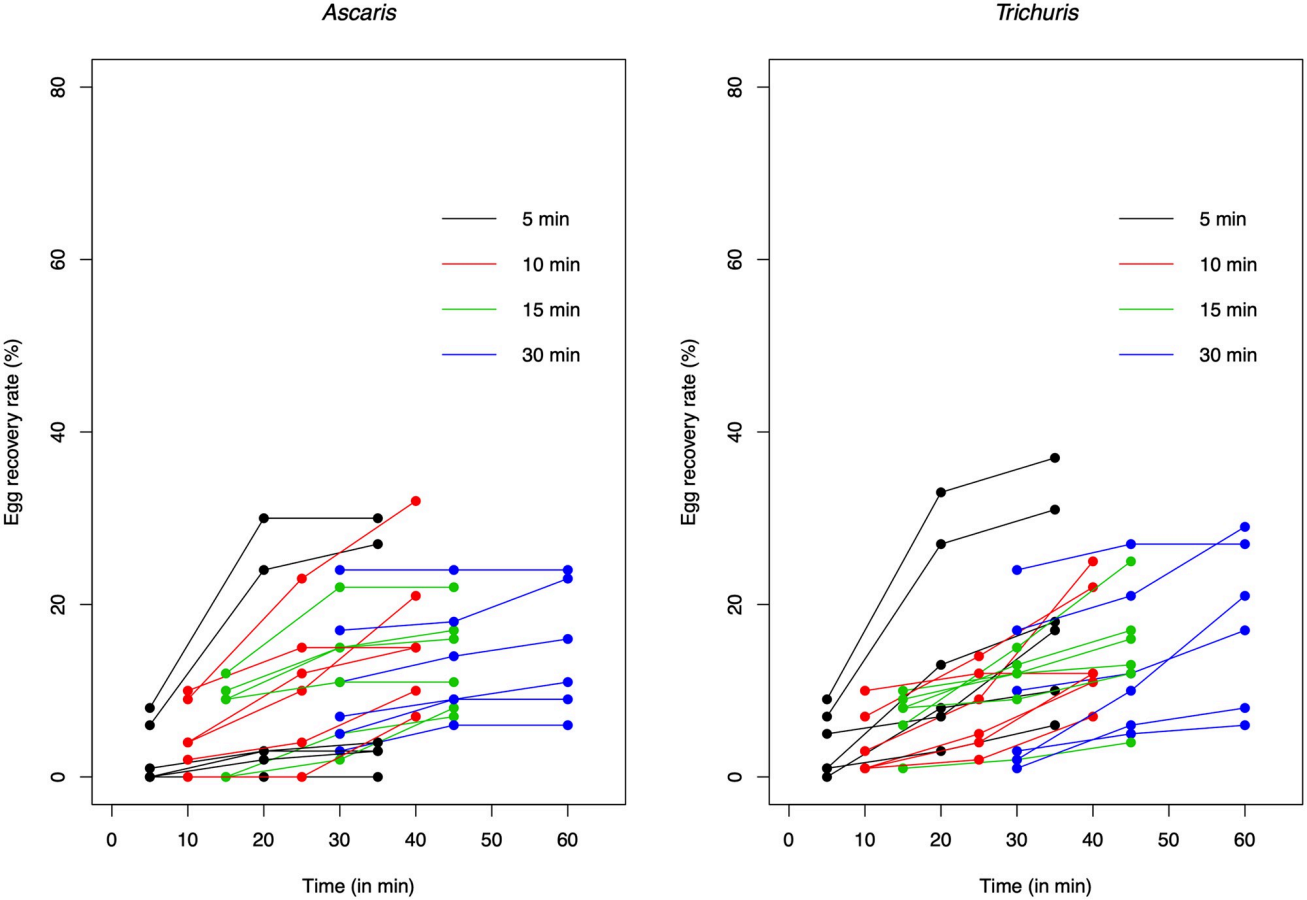
The recovery rate of *Ascaris* and *Trichuris* eggs following serial flotation. The spaghetti plots illustrate the cumulative egg recovery rate when serial flotation was applied. The color code differentiates the time at which the first coverslip was removed from the flotation tube.

Table 1 and Fig 2 provide a summary of the experiments designed to optimize our straining-flotation method. Using ZnSO_4_ resulted in a significantly (*p* ≤0.05 /3 pair-wise comparisons) higher egg recovery rate compared to both NaCl (W = 6.5, *p* = 0.009) and MgSO_4_ (W = 3.5, *p* = 0.003) for *Ascaris*, but not for *Trichuris*. For this STH, none of the pair-wise comparisons indicated a significant (*p* >0.05 /6 pair-wise comparisons) difference across the different FS. The recovery of *Ascaris* and *Trichuris* eggs increased when the cover slip was kept for longer on the test tube. Although the pair-wise comparison did not reveal any significant differences (*p* >0.05/six pair-wise comparisons) in egg recovery rate based on the first coverslip, a flotation period of 30 min did not result in any zero egg counts across both STH species (Fig 2). This was in sharp contrast with the other flotation periods where zero egg counts were observed, particularly for *Ascaris*. For this STH, zero egg counts were observed in 3, 1 and 2 out of the six replicates for a flotation period of five, 10, and 15 min, respectively. For *Trichuris*, zero egg counts were only observed in one replicate for the flotation period of five min.

**Table 1:** The egg recovery rate for *Ascaris* and *Trichuris* across five experiments. The *p*-values were based on a non-parametric test (Wilcoxon rank sum test).

Experiment	*Ascaris*		*Trichuris*	
	Median egg recovery rate (%) (minimum; maximum)	*p*-value pairwise comparison	Median egg recovery rate (%) (minimum; maximum)	*p*-value pairwise comparison
***Flotation experiment (type of flotation solution)***				
MgSO_4_ (n = 8)	7.5 (0; 21.0)	NaCl: 0.430 ZnSO_4_: 0.003	16.5 (4; 25)	NaCl: 0.958 ZnSO_4_: 0.226
NaCl (n = 8)	10.5 (3.0; 17.0)	ZnSO_4_: 0.009	14.0 (6; 25)	ZnSO_4_: 0.100
ZnSO_4_ (n = 8)	23.5 (11.0; 32.0)		20.0 (12; 37)	
***Flotation experiment (period of flotation for 1***^***st***^ ***coverslip)***				
5 min (n = 6)	0.5 (0.0; 8.0)	10 min: 0.253 15 min: 0.157 30 min: 0.044	3.0 (0; 9)	10 min: 0.868 15 min: 0.145 30 min: 0.227
10 min (n = 6)	4.0 (0.0; 10.0)	15 min: 0.625 30 min: 0.128	2.0 (1; 10)	15 min: 0.220 30 min: 0.252
15 min (n = 6)	9.0 (0.0; 12.0)	30 min: 0.470	8.0 (1; 10)	30 min: 0.809
30 min (n = 6)	9.0 (3.0; 24.0)		6.5 (1; 24)	
***Flotation experiment (total period of flotation over three coverslips)***				
35 min (n = 6)	3.5 (0; 30)	40 min: 0.228 45 min: 0.378 30 min: 0.378	17.5 (6; 37)	40 min: 0.688 45 min: 0.423 30 min: 0.810
40 min (n = 6)	15.0 (7; 32)	45 min: 0.872 60 min: 0.936	12.0 (7; 25)	45 min: 0.747 60 min: 0.688
45 min (n = 6)	13.5 (7; 22)	60 min: 0.810	14.5 (4; 25)	60 min: 0.423
60 min (n = 6)	13.5 (6; 24)		19.0 (6; 29)	
***Centrifugal force experiment***				
1,000 *g* (n = 8)	28.0 (17.0; 55.0)	0.293	22.0 (10; 41)	0.753
2,000 *g* (n = 8)	48.0 (15.0; 55.0)		24.0 (14; 44)	
***Tween experiment***				
0% (n = 8)	48.0 (15.0; 55.0)	0.371	24.0 (14.0; 44.0)	0.010
0.05% (n = 8)	37.0 (28.0; 46.0)		39.5 (30.0; 57.0)	
***Volume soil experiment***				
1 mL (n = 12)	49.5 (13.0; 77.0)	0.665	27.5 (3; 42)	0.665
3 mL (n = 12)	51.5 (27.5; 69.0)		27.5 (6; 55)	
***Strainer experiment***				
No (n = 8)	66.5 (53.0; 80.0)	0.674	33.5 (20; 57)	0.103
Yes (n = 8)	65.5 (46.0; 78.0)		21.0 (10; 51)	

The results of the serial flotation examination of the samples indicated that repeating the flotation process increased the final egg recovery rate for both *Ascaris* and *Trichuris*, and this regardless of the period of flotation of the first cover slip. There was no significant (*p* >0.05/6 pair-wise comparisons) difference in the total number of eggs recovered between a serial flotation of 35 min (= 5 + 15 +15), 40 min (= 10 + 15 +15), 45 min (= 15 + 15 +15) and 60 min (= 30 + 15 + 15).

The median egg recovery rates in the remaining laboratory experiments did not reveal major differences in egg recovery rate. A difference in median egg recovery rates of more than 10-point percent was observed between the centrifugal forces for *Ascaris* (2,000 *g*: 48.0% *vs*. 1,000 *g*: 28.0%), the Tween 80 concentrations for both *Ascaris* (Tween: 37.0% *vs*. no Tween: 48.0%) and *Trichuris* (Tween: 39.5% *vs*. no Tween: 24.0%), and when the serial flotation step was replaced by an additional straining step for *Trichuris* (no straining: 33.5% *vs*. straining: 21.0%). The non-parametric test confirmed that adding Tween resulted in a significantly improved egg recovery rate (W = 8, *p* = 0.010) for *Trichuris*. In all other comparisons, the test did not reveal any significant difference in egg recovery rate (*p* >0.05).

Based on the outcome of the experiments we decided to use ZnSO_4_ as FS, to centrifuge the tubes at 2,000 *g* and to replace the repeated flotation step by an additional straining step. Concerning the Tween, we decided not to add the Tween into FS, rather we added 0.5% Tween 80 to the soil to facilitate homogenization of the eggs/larvae prior to sieving. Fig 1 provides a schematic overview of our optimized in-house straining-flotation method.

### Diagnostic performance of the optimized straining-flotation method

**Fig 3:**
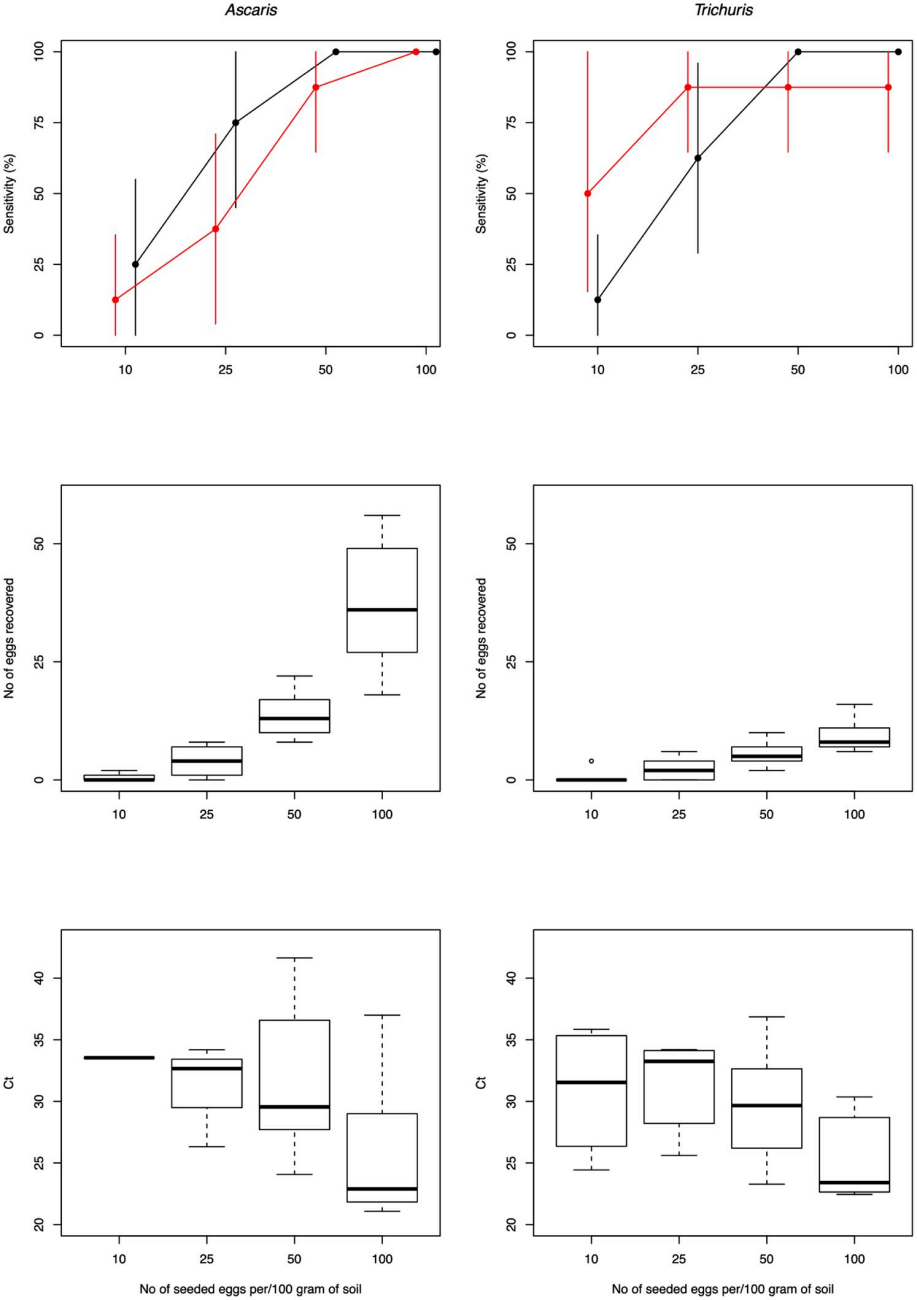
The diagnostic performance of the optimized straining-flotation method to detect and quantify *Ascaris* and *Trichuris* eggs in soil. The panels describe the sensitivity for both microscopy and qPCR (**top row panels**), the egg recovery rate for microscopy (**middle row panels**) and the Ct-values for qPCR (**bottom row panels**) separately for *Ascaris* (**left column panels**) and *Trichuris* (**right column panels**).

Combining both microscopy and qPCR resulted in an overall sensitivity of 81.3% for *Ascaris* and 87.5% for *Trichuris*. The outcome of the different experiments is graphically summarized in Fig 3. S1 Table provides a numeric summary of the diagnostic performance. For microscopy, the sensitivity was 100% when the concentration was 50 eggs per 100 grams of soil for both STHs. For a concentration of 25 eggs per 100 grams of soil, the sensitivity was 75.0% (95%CI: 45.0; 100) for *Ascaris* and 62.5% (95CI: 29.9; 96.0) for *Trichuris*, while for a concentration of 10 eggs/100 grams of soil the sensitivity was 25.0% (95%CI: 0.0; 55.5) and 12.5% (95%CI: 0.0; 35.4), respectively. Given the observed recovery rate in the experiments designed to optimize the straining-flotation method, it did not come with a surprise that the estimated egg concentration is underestimating the true underlying egg contamination. Yet, it is important to note that the egg recovery rate did not remain unchanged across the different levels of egg contamination for *Ascaris* but increased as a function of increasing egg contamination. For *Trichuris*, the egg recovery rates remain largely unchanged. Generally, qPCR was less sensitive than microscopy for *Ascaris* (75.0% *vs*. 59.4%). For *Trichuris*, qPCR was more sensitive in a higher detection rate when concentration was low (10 eggs: 50.0% *vs*. 12.5%; 25 eggs: 87.5% *vs*. 62.5%) but resulted in a lower rate when concentration was high (50 eggs: 87.5% *vs*. 100%; 100 eggs: 87.5% *vs*. 100%).

### Application of the optimized straining-flotation method on field samples

The analysis of the 399 field samples (schools: 170; households: 199; markets: 30) with both microscopy and qPCR revealed the presence of 8 different helminth species of medical importance, including but not limited to the human STHs. The most prevalent helminths were *Ascaris* (65.7%) and *Taenia saginata* (52.4%), followed by *Trichuris* (21.6%). The other helminths were only observed in a minority of the cases (*Enterobius*: 5.8%; *Hymenolepis*: 5.3%; *Strongyloides*: 1.5%; *Necator*: 0.8%; *Schistosoma*: 0.8%). A numerical overview of environmental contamination for all helminth species at the different school compounds, households and open markets is provided in S2–S4 Tables. In the following paragraphs we will focus on the environmental contamination of STHs only.

#### The environmental STH contamination at school compounds

**Fig 4:**
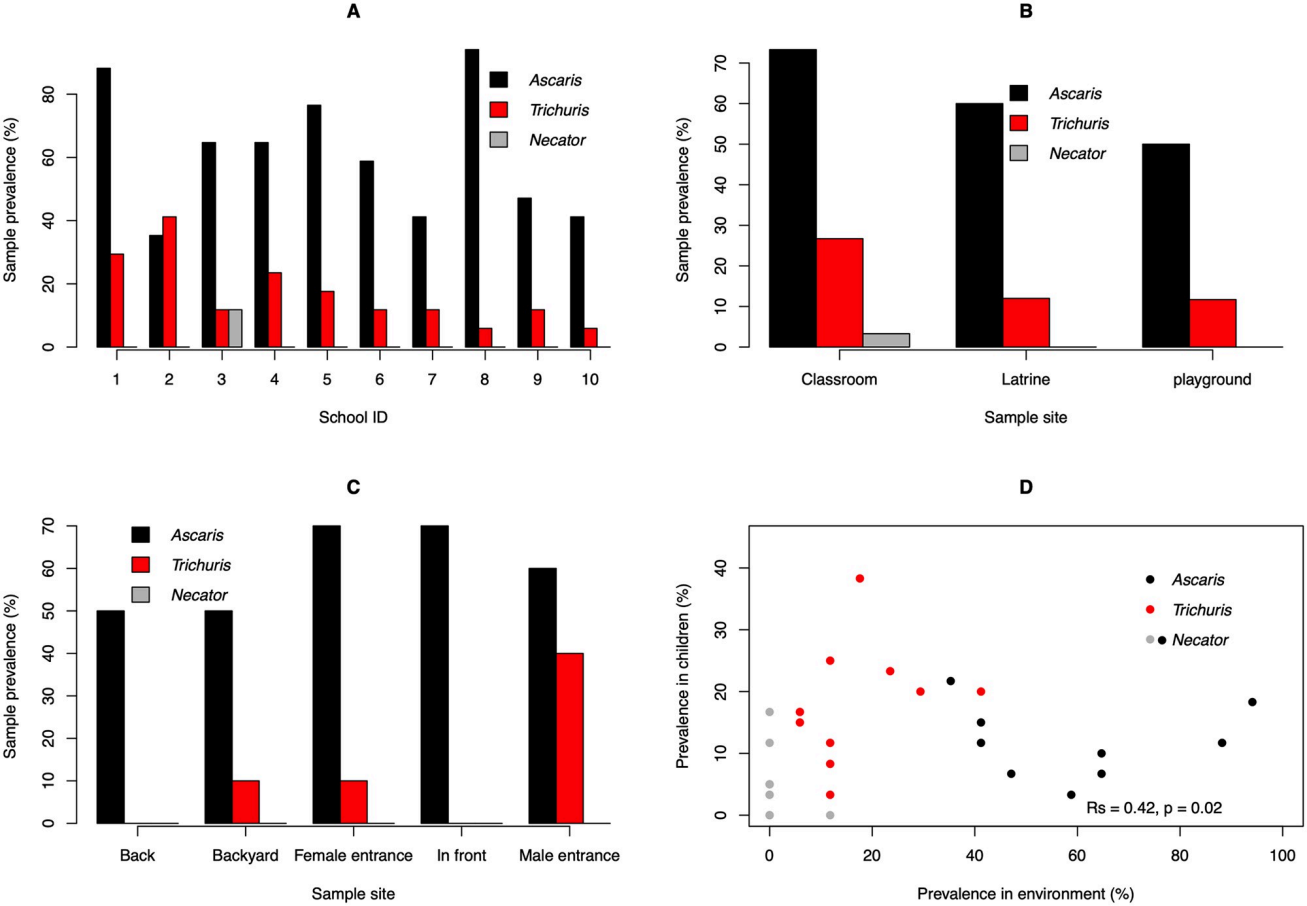
The environmental STH contamination at 10 school compounds, Jimma Town (Ethiopia). This figure illustrates the environmental soil-transmitted helminth contamination across 10 governmental schools in Jimma Town, Ethiopia (**Panel A**), at different sites within schools (classroom, latrine and playground; **Panel B**), around different places around the school latrine (**Panel C**), and the association between prevalence of STH infections in children and the proportion of the samples contaminated with STH at the school level (**Panel D**).

The environmental contamination with human STHs at school compounds is illustrated in Fig 4. Overall, human STHs were observed in 113 out of the 170 (66.5%) samples that were examined by both microscopy and qPCR. *Ascaris* was the most observed STH species (61.2%), followed by *Trichuris* (17.1%). Of the two human hookworms *N*. *americanus* was observed in 2 samples (1.2%) only. Both *Ascaris* and *Trichuris* were found across all ten schools, the sample prevalence ranging from 35.3% to 94.1% for *Ascaris*, and from 5.9% to 41.2% in school #6 for *Trichuris*. *N*. *americanus* was only observed in school #1 (11.8%) (Fig 4A).

When focusing on the different sampling sites within the school compounds (Fig 4B), the sample prevalence of any human STH was the highest for classrooms (81.6%), followed by both latrines (62.0%) and playground (55.0%). The sample prevalence for each of the three STHs followed a similar pattern, sample prevalence being highest for classrooms, followed by latrines and playground (*Ascaris*: 73.3% *vs*. 60.0% *vs*. 50.0%; *Trichuris*: 26.7% *vs*. 12.0% *vs*. 11.7%; *N*. *americanus*: 3.3% *vs*. 0.0% *vs*. 0.0%). Around the latrines (Fig 4C), the sample prevalence of any human STH was at least 50% and did not vary across the different sampling sites (behind the latrine: 50.0%; the back yard of the latrine: 50.0%, in front of the latrine: 70.0%; the entrance for girls: 70.0%; the entrance for boys: 70.0%). While for *Ascaris*, the sample prevalence did not considerably vary across the sampling sites (50.0% to 70.0%), the presence of *Trichuris* was not observed in samples from both behind and in front of the latrine. The sample prevalence at both the entrance for girls and the backyard of the latrine equaled 10.0%, and at the male entrance *Trichuris* was found in 40.0% of the samples. The association between prevalence of STH infections in children and the proportion of the samples contaminated with STH at the school level is illustrated in Fig 4D, highlighting that the prevalence of any infections in school children was significantly associated with the proportion of environmental samples containing any STH life stages (Rs = 0.42, *p* = 0.02). S5 Table provides an overview of the STH infections across school children in the 10 schools in Jimma Town, Ethiopia.

#### The environmental STH contamination at households

**Fig 5:**
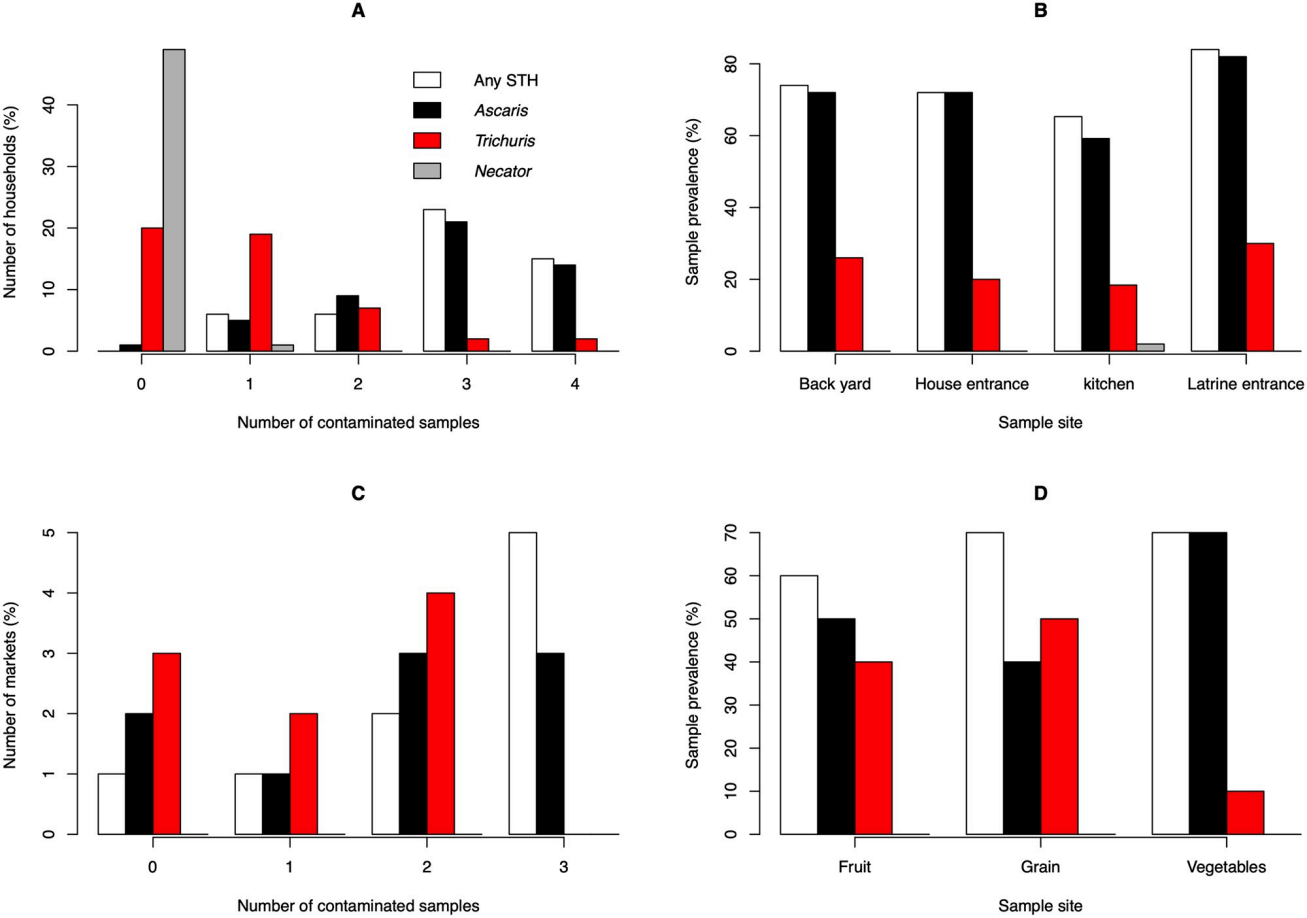
The environmental contamination with human STHs at the households and open markets. This figure illustrates the environmental soil-transmitted helminth contamination across 50 households (Panels A and B) and 9 open markets (Panels C and D).

The environmental contamination with human STHs at the households is illustrated in Fig 5. Overall, human STHs were observed in 147 out of the 199 (73.9%) samples that were examined by both microscopy and qPCR. Similarly, as for the school compounds, *Ascaris* was the most observed STH species (71.4%), followed by *Trichuris* (23.6%). *N*. *americanus* was again observed in 1 sample (0.5%). With exception of one, *Ascaris* was found in all households, *Trichuris* was found in 60.0% of the households (Fig 5A). *Necator* was only observed in one household. The contamination varied across the different sampling sites (Fig 5B). Generally, samples collected at the latrine entrance were most contaminated (any STH: 84.0%; *Ascaris*: 82.9%; *Trichuris*: 30.0%). The least contamination was observed in the kitchen (any STH: 65.3%; *Ascaris*: 59.2%; *Trichuris*: 18.4%).

Table 2 summarizes the characteristics of the different households and the corresponding environmental contamination measured by the mean STH egg counts across the samples. On average, 5.5 people lived in the households. In 12 out of the 50 households, at least one child <3 years of age was reported and in 40 households (80.0%) animals (e.g. dogs, cats, chicken and cattle) were observed. With only a few exceptions (n = 2; 4.0%), all households had a latrine. Most of the latrines were open well (n = 34; 68.0%), all others were pit latrines (n = 14; 28.0%). Half of the latrines did not have a wall (n = 26; 52.0%) or a roof (n = 25, 50.0%). In one third of the cases, there was no door (n = 15; 30.0%). Flies were observed around the latrines in 34 households (68.0%), and excreta were visible in six households (12.0%). The 20% of the household heads reported that their children practiced open field defecation. Almost all visited households (98.0%) did not have water in the latrine and 46.0% did not have any water supply at their household compounds. Most of the kitchens (n = 36, 72.0%) had walls and a floor of soil, and 11 households had an open space kitchen (n = 11, 22.0%). In only in a few cases (n = 3; 6.0%), we observed a kitchen with walls and a concrete floor. The mean distance from the latrine entrance to the house entrance was 16.7 m (ranging from 4 to 35 m). Given the high prevalence of STHs at the household (presence of STH eggs/DNA was observed in at least 2 samples in 43 out of 49 households), we did not explore any associations between presence of environmental contamination at the household level and the household characteristics and the infection status of the child. There were no household characteristics that explained the differences in eggs in 100 grams of soil (*p*-value >0.05). The median (minimum; maximum) environmental contamination in the households of the children with STH infections equaled 10.5 eggs per 100 grams of soil (0.5; 1,153.0) and was not significantly (*p* = 0.453) different from the households of children without STH infections (6.5 (0.0; 441.5)).

**Table 2:** The characteristics and the corresponding environmental contamination of 50 households in Jimma Town, Ethiopia. The environmental contamination measured by the mean soil-transmitted helminth egg counts across the samples. The *p*-value was based on the Wilcoxon rank sum test in case of two-level variables, while in case of variables with more than two levels it was based on the Kruskall Wallis test.

Variable	N (%)	Median STH egg count (minimum; maximum	*p*-value
Presence of child <3years old			
Yes	12 (24.0)	12.8 (1.5; 441.5)	0.400
No	38 (76.0)	9.0 (0.0; 1,153.0)	
Presence of animal (dog, cat, hen and cow)			
Yes	40 (80.0)	12.8 (5.5; 34.0)	0.149
No	10 (20.0)	7.5 (0.0; 1,153.0)	
Presence of a latrine			
Yes	48 (96.0)	9.8 (0.0; 1,153.0	0.921
No	2 (4.0)	221.0 (0.5; 441.5)	
Type of latrine			
No latrine	2 (4.0)	221.0 (0.5; 441.5)	0.826
Pit latrine	14 (28.0)	7.8 (0.0; 34.0	
Open well	34 (68.0)	10.0 (0.0; 1,153.0)	
Wall around latrine			
No latrine	2 (4.0)	221.0 (0.5; 441.5)	0.943
Yes	26 (52.0)	10.3 (0; 49.0)	
No	24 (48.0)	9.5 (1.0; 1,153.0)	
Latrine door			
No latrine	2 (4.0)	221.0 (0.5; 441.5)	0.986
Yes	15 (30.0)	10.5 (0; 34.0)	
No	33 (66.0)	9.5 (0; 1,153.0)	
Latrine roof			
No latrine	2 (4.0)	221.0 (0.5; 441.5)	0.799
Yes	25 (50.0)	10.0 (0.0; 42.5)	
No	23 (46.0)	9.5 (1.0; 1,153.0)	
Flies around latrine			
No latrine	2 (4.0)	221.0 (0.5; 441.5)	0.923
Yes	32 (64.0)	9.8 (0.0; 1,153.0)	
No	16 (32.0)	8.3 (0.0; 34.0)	
Visible stool observed on household areas			
Yes	6 (12.0)	9.5 (0; 1,153.0)	0.881
No	44 (88.0)	10.3 (2; 14.5)	
Defecation outside latrine			
Yes	12 (24.0)	8.0 (0.5; 441.5)	0.503
No	38 (76.0)	9.8 (0.0; 1,153.0)	
Water supply at home			
Yes	27 (54.0)	10.0 (0.0; 1,153.0)	0.311
No	23 (46.0)	9.5 (0; 441.5)	
Water in the latrine			
No latrine	2 (4.0)	221.0 (0.5; 441.5)	0.342
Yes	1 (2.0)	34.0 (34.0; 34.0)	
No	47 (94.0)	8.25 (0.5; 16.0)	
Type of kitchen			
Open space	11 (22.0)	9.5 (0.0; 30.5)	0.607
Soil floor with wall and roof	36 (72.0)	10.3 (0; 1,153)	
Cement floor with wall and roof	3 (6.0)	5.0 (3.5; 34.0)	

#### The environmental contamination with human STHs at the open markets

The environmental contamination with human STHs at the open markets is illustrated in Fig 5C and 5D. Overall, human STHs were observed in 20 out of the 30 samples that were examined by both microscopy and qPCR. Here too, *Ascaris* was the most observed STH species (53.3%), followed by *Trichuris* (33.3%). *Necator* was not observed in any of the markets. *Ascaris* was detected at seven out of the nine open markets and *Trichuris* at six open markets. At most open markets, at least two out of the three (eight markets) or six soil samples (one large open market) were contaminated with any STHs (seven out of nine markets) and *Ascaris* (six out of nine markets). For *Trichuris*, majority of the markets (n = 5) had less than two contaminated samples. There was little variation in contamination with any STHs across the locations where fruit (60.0%), grains (70.0%) and vegetables (70.0%) where sold. Yet, on areas where vegetables were sold, mainly *Ascaris* (70.0%) was observed and to a lesser extent (*Trichuris*). At the other areas, both STHs were found at a similar rate.

#### Comparison of microscopy and qPCR

When the optimized method was applied to field samples, eggs and/or DNA of *Ascaris* and *Trichuris* was detected in 262 and 86 field samples, respectively. Assuming a perfect specificity for both microscopy and qPCR, the sensitivity of microscopy was 77.9% for *Ascaris* and 38.4% for *Trichuris*. Compared to microscopy, qPCR was less sensitive for *Ascaris* (61.0%) and more sensitive for *Trichuris* (74.4%). As illustrated in S1 Fig, the Ct-value dropped as a function of increasing numbers of eggs counted by microscopy for *Ascaris* and to a lesser extent for *Trichuris*. For the other helminths of medical importance, qPCR outcompeted microscopy. For example, based on the results of microscopy and qPCR combined *Taenia* was observed in 209 samples, of which 99.5% were detected by qPCR and 6.2% by microscopy. Similar findings were observed for *Enterobius* (23 cases, of which 91.3% by qPCR and 8.7% by microscopy). For *Hymenolepis*, microscopy was more sensitivity (22 cases, of which 40.9% by qPCR and 59.1% by microscopy). For the remaining helminth species, a comparison was difficult as morphological differentiation was not straight forward (*Strongyloides* and hookworms) or due to the low number of cases (*Schistosoma*).

### KAP towards STHs

A total of 422 school children participated in this questionnaire. The age of the children ranged from 12 to 18 years (median = 14), and the ratio boys to girls equaled 1:1.21. Half of the children were Muslim (51.9%), the other half was Christian (48.1%). The education of the parents was poor in majority of the cases. More than half of the fathers did not complete senior high school (illiterate: 13.7%; elementary school: 25.8%; junior high school: 18.0%) and half of the mother did not complete junior high school (illiterate: 28.0%; elementary school: 30.6%). Majority of the fathers were either farmer (n = 125; 29.6%) or employees of the government (n = 122; 28.9%), while mothers were mainly a housewife or a cleaner (n = 287; 68.0%).

Generally, most of children were aware of STHs, 372 (88.2%) children confirming that they had heard the STH-related words, such as ‘intestinal worms’, ‘*Ascaris’*, ‘*Trichuris’* and ‘*hookworms’*, before. This was much in line with other major diseases (HIV: 98.3%; malaria: 91.0% and tuberculosis: 82.0%). However, important differences across the words were observed. For example, ‘intestinal worms’ and ‘*Ascaris’* were known by approximately 90% of the children. The words ‘hookworms’ and ‘*Trichuris*’ were only known by 12.6% and 0.9% of the children, respectively. Teachers were the main source of information (88.9%), followed by television (73.7%) and radio (63.3%). Children also heard about the aforementioned words through nurses and doctors (26.8%) and health extension workers (25.8%). On the impact of STHs on the human body, majority confirmed that STHs cause stomach-ache (n = 384; 91.0%) and that they do share food of the host (n = 315, 74.6%). Approximately half of the students (47.2%) did not know that STHs (hookworm) also suck blood. A minority of the children falsely declared that STHs cause blood in the urine (19.7%) and blindness (10.4%). Many students (82.9%) knew that adult STHs are dwelling in the intestines, but there was also a minority of the children that thought that adult STHs can be found in other parts of the human body (skin: 15.2%, lung: 13.0%, heart: 7.1%, head: 5.9% and eyes: 4.0%). Only few students (n = 26, 6.2%), did not know that doctors and nurses can provide drugs against STH infections. In addition, a considerable part of the students (n = 114, 27.0%) incorrectly answered that there is a vaccine against STH infections. Although, most of the students know that STHs are not transmitted through air (n = 95, 22.5%), insect bites (n = 58, 13.7%), blood (n = 64, 15.2%) and saliva (n = 27, 6.4%), only 173 children (41.0%) confirmed that STHs are transmitted through stool. Many children, 327 (77.5%) reported that STH is transmitted by eating raw meat. Children usually defecate at the latrine of their home 64.0% (n = 270), followed by the latrine in the compound (village common latrine; n = 145; 34.4%). Only few students defecate in the latrines of the school (0.7%) and in the bush (0.9%). Majority of the children reported that they always washed their hands before eating food (n = 400; 94.8%) and after using the latrine/toilet (n = 361; 85.5%). Washing soap was often used (n = 255; 60.4%). From all the interviewed students, 18.5% (n = 78) did not trim their fingernails. Most of the children (n = 292; 69.2%) trimmed their fingernails once a week, 11.1% (n = 47) once every two weeks and 1.7% (n = 7) less than one time per month. Almost all students (n = 419; 99.3%) wore shoes, but 21.8% (n = 92) of them did not always do this. From all the study participants, 33.1% (n = 135) did not worry about the risk of getting an STH infection and 11.9% (n = 49) would not be anxious if they got infection with intestinal worms.

## Discussion

Large-scale deworming programs have been successful in reducing the disease burden attributable to STH infections, but re-infection in the absence of other intervention measures is unavoidable. Today, it remains unclear where and how (re-)infection occurs, which further impedes the development of targeted and perhaps more cost-effective control strategies. In the present study, we aimed to identify possible sources of infection by assessing the environmental contamination in school compounds, households and open markets. To this end, we first performed a series of laboratory experiments designed to optimize an in-house straining-flotation method to detect and quantify *Ascaris* and *Trichuris* eggs in soil and to validate the diagnostic performance of the optimized method when followed by both microscopy examination and qPCR. In a second phase, we applied this method to assess the level of STH contamination in environmental samples collected from school compounds, households and open markets in an area that is endemic to STHs. Subsequently, we explored associations between the environmental contamination and both the corresponding STH epidemiology at the level of the schools and the household characteristics. Finally, we assessed the KAP towards STH among school children.

### Our soil straining-flotation method has a moderate diagnostic performance

Our experiments were designed to optimize our in-house soil straining-flotation method indicated that (i) ZnSO_4_ (specific density = 1.35), (ii) 30 min of flotation, (ii) increasing centrifugal force and (iii) examination of multiple coverslips following repeated flotation significantly improved the recovery of *Ascaris* and *Trichuris* eggs. These findings were not unexpected and are in line with previous studies that assessed the impact of both the specific density and the nature of FS [69,70], and repeated flotation [25]. In our final standard operating procedure (SOP; Fig 1), we replaced the repeated flotation step by an additional straining step to reduce the sample process time to 30 min per sample when samples were examined by microscopy. Generally, the median reported time in literature equaled 2.0 h (ranging from <1 h to 2 days) [58], highlighting that our method allows for a moderately high sample throughput.

For microscopy, the analytical sensitivity (= concentration of eggs for which a positive test result was obtained in at least 95% of the replicates) of our final SOP was 50 eggs per 100 grams of soil (or 1 egg in 2 grams of soil) for both STHs (*Ascaris* and *Trichuris*). The median egg recovery rate for 100 eggs per grams of soil was 36.0% for *Ascaris* and 8.0% for *Trichuris*. For qPCR, the analytical sensitivity was 100 eggs per 100 grams of soil for *Ascaris*, while for *Trichuris* this was >100 eggs per 100 grams of soil. This difference in analytic sensitivity might be due to a difference in the amount of soil that was analyzed by microscopy and qPCR. While we analyzed all soil that was collected from the 20 μm strainer with microscopy (**step 9** in Fig 1), only half of this was analyzed with qPCR. Processing half of the material was a strategic choice to have a back-up if the sample process failed.

Although a head-to-head comparison with other studies is difficult to impossible due the diversity in SOPs and different helminth species (see Introduction), two important aspects can be noted. First, we stress-tested our method in a range of egg counts (0.1 to 1 egg per gram of soil) and mass of soil (100 grams) that have been rarely examined. Indeed, based on a review of the literature the median egg concentration was 20.0 eggs per gram of soil (ranging from 4 to 32,393) for *Ascaris* [58]. For *Trichuris*, the egg concentration ranged from 1 to 32,393 eggs per gram of soil for *Trichuris* (only two studies were reported in this review; [58]). Compared to the few studies available, our method revealed to have a low analytic sensitivity. For example, spiking experiments with *Toxocara canis* eggs (1, 10, 25, 50, 100, and 200) into one gram of soil [69], revealed that at least one egg was microscopically detected in replicates with 10 eggs, resulting in an analytical sensitivity not lower than 10 eggs per gram soil (*vs*. 0.5 eggs per gram of soil in the present study). Second, the recovery rate of our method revealed to be within the range reported for *Ascaris* (median egg recovery rate reported by [58] equals 25.0%, ranging from 9.5 to 82%), but at the lower end for *Trichuris* (only two studies were reported in this review; 80.0% and 96.4%; [58]). Although, one cannot rule out variation in soil type or SOPs, this poor egg recovery rate for *Trichuris* can also be explained by the mesh size we used to strain the soil samples (25 μm). *Trichuris* eggs measure size 50–55 μm x 20–25 μm [71], and therefore they may pass the sieve resulting in the observed poor egg recovery rate. Including a sieve with a mesh size of 20 μm would be an obvious modification of our SOP.

Although the aforementioned paragraphs highlight that our method allows for a moderately high throughput and has a moderate diagnostic performance, the purchase of the ‘Vibratory Sieve Shaker’ (with the corresponding sieves) is a substantial investment (8,000 EURO, incl 17% VAT). In addition to this, the access of constant flow of relatively large volumes of water, was also perceived as an important obstacle.

### Life stages of helminths of medical important helminths are ubiquitous in the environment

Our result highlights that the presence of 8 different helminth species of medical importance, including but not limited to the human STHs. The most ubiquitous helminths were *Ascaris* (65.7%) and *Taenia saginata* (52.4%), followed by *Trichuris* (21.6%). The other helminths were only observed in a minority of the cases (*Enterobius*: 5.8%; *Hymenolepis*: 5.3%; *Strongyloides*: 1.5%; *Necator*: 0.8%; *Schistosoma*: 0.8%). Generally, both the presence and the prevalence of these helminths in the environment are reflecting the infection rates of school children [59,60] and adults [60], except for *Taenia*. For this helminth, the infection rates 2.3% [72–73] in Ethiopia (including our study area) are lower than when one would expect based on the environmental contamination. This might be explained by both the poor sensitivity of the method used to diagnose infections (Kato-Katz thick smear: 52% (95% credible interval [7–94]) *vs*. qPCR sensitivity 94% (95% credible interval [88–98]) [74] and the number of eggs excreted in the environment once a gravid proglottid has actively left the host (one gravid proglottid may contain up to 2,000 eggs [75]).

The environmental STH contamination was high across all major sample locations, households being the most contaminated (73.9% *vs*. ~66% in both open markets and school compounds). The contamination level at school compounds was exceeding that previously reported (Nigeria: 53.6% [76] and 32.0% [77]; Cameroon: 17% [78]; Ethiopia: 11.3% [79]). The contamination at households was comparable to that observed in a study in Bangladesh (78.0% [24]) but higher that of a study in Kenya 26.8% [25]. The contamination level in the present study was higher than that previously reported (15% in Cameron [80]).

Although a head-to comparison remains once more difficult, this higher prevalence STH in our surveys could be due to the higher amount of soil (100 grams of soil) and combining both microscopy and qPCR in the sample process. The latter method allowing for a more sensitive (though not for all helminth species), specific (e.g., speciation of the hookworm and *Taenia* species) and simultaneous detection of multiple helminth species [55,56]. Although these results indicate that helminths are ubiquitous in the environment, we did not assess the viability of the different life stages. Viability can be assessed by specific dyes [81,82]. Because of this, we are not able to accurately estimate the risk of infection.

### Are schools a source of STH infections in children or are children contaminating schools?

There was an association between prevalence of STH infections in children and the proportion of samples contaminated with STHs at the school level (see Fig 5D). Yet, it remains unclear whether schools are a source of STH infections or whether children are contributing to school contamination. In either way, the fact that the contamination in classrooms is higher than around latrines is remarkable, yet not unexpected. This is because, the inside of the latrines was extremely contaminated with human stool. As a consequence of this, it is not unlikely that children step into human stool when using the latrines and stepping into the classroom with shoes contaminated with stool. Moreover, while life stages surrounding the latrines might be washed away during episodes of rain, this is not likely to occur in the roofed classrooms. Lack of awareness on route of STH transmission and prevention methods among study participants further highlight the need for health education and improved WASH intervention in the schools to minimize both environmental contamination and transmission of STH.

### Other limitations of the study

This study has a number of other limitations. First, while the environmental samples were collected during both the dry and wet season, we did not dry the samples prior to processing them with our in-house straining flotation method. This might be important to for future studies designed to assess seasonal differences in environmental contamination. Second, to gain more insights in the role of children in contaminating the school, it would have been important to verify an association both between children defecating at the school and the environmental contamination found at schools and between defecating at school and the STH infection status. Although we indeed capture the defecating behavior in the KAP survey, due to time constrains (survey was conducted shortly before school holidays) it was assessed in five out of ten schools only. Moreover, the children were unexpectedly dewormed as part of the national STH control program while the KAP survey was conducted, and hence we were not able to verify associations between their KAP towards STH and their infection status. Finally, the validation of our in-house straining-flotation method was limited to *Ascaris* and *Trichuris* only. It would be important to also assess this for the other helminth species.

## Conclusions

Our optimized straining-flotation method has a moderate diagnostic performance and revealed that life stages of helminths are ubiquitous in the environment, reflecting both the poor sanitary facilities at the households and schools, and the lack of awareness of STHs. Further research is required to gain more insights into the contribution of these life stages to transmission.

## Supporting information

S1 Fig The association in contamination in environmental samples measured by microscopy and qPCR. These scatterplots represent the association in *Ascaris* and *Trichuris* contamination measured by microscopy (in eggs per 100 grams of soil) and qPCR (in Ct). A Ct-value of zero indicates absence of DNA.
(TIF)

S1 Table The ability of the optimized straining-flotation method to detect and quantify STH eggs in soil. The egg counts were adjusted for the amount of sample examined. The Ct-value summarized in this table only reflect those that were observed in qPCR-positive samples only.
(DOC)

S2 Table Helminth contamination of soil samples collected in 10 school compounds in Jimma Town, Ethiopia. (DOC)

S3 Table Helminth contamination of soil samples collected from 50 households at Jimma Town, Ethiopia. (DOC)

S4 Table Helminth contamination of soil samples collected from 9 markets at Jimma Town, Ethiopia. (DOC)

S5 Table Soil-transmitted helminths. infections in 10 schools in Jimma Town, Ethiopia. (DOC)

S1 Info The questionnaire to gain insights into the characteristics of the households. (DOC)

S2 Info The questionnaire on knowledge, attitude and practice towards soil-transmitted helminths. (DOC)

S1 Data The optimization and the diagnostic performance of the soil straining-flotation method. (XLSX)

S2 Data The environmental contamination at school compounds, households and open markets. (XLSX)

S3 Data The household characteristics. (XLSX)

S4 Data The knowledge, attitude and practice towards soil-transmitted helminths. (XLSX)
