# Supplementary figures and images for: Assessment of environmental contamination with soil-transmitted helminths life stages at school compounds, households and open markets in Jimma Town, Ethiopia
Source: PLoS Negl Trop Dis. 2022 Apr 4;16(4):e0010307. doi: 10.1371/journal.pntd.0010307 (PMC9009776; doi:10.1371/journal.pntd.0010307)

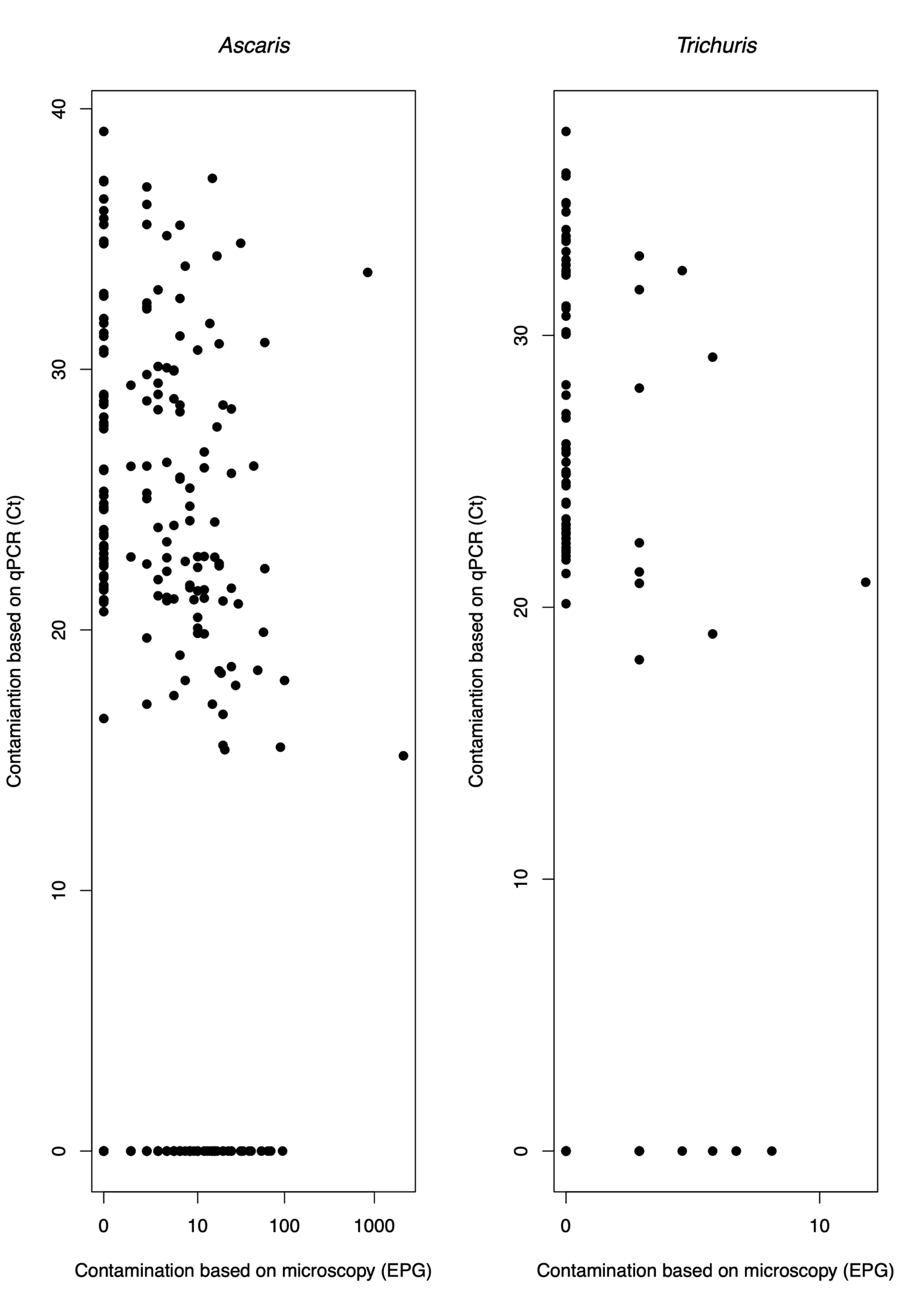

Supplement: S1 Fig — These scatterplots represent the association in Ascaris and Trichuris contamination measured by microscopy (in eggs per 100 grams of soil) and qPCR (in Ct). A Ct-value of zero indicates absence of DNA. (TIF) [file pntd.0010307.s001.tif]
