# Supplementary material for: Assessment of environmental contamination with soil-transmitted helminths life stages at school compounds, households and open markets in Jimma Town, Ethiopia
Source: PLoS Negl Trop Dis. 2022 Apr 4;16(4):e0010307. doi: 10.1371/journal.pntd.0010307 (PMC9009776; doi:10.1371/journal.pntd.0010307)
Supplement: S1 Table — The egg counts were adjusted for the amount of sample examined. The Ct-value summarized in this table only reflect those that were observed in qPCR-positive samples only. (DOC) [file pntd.0010307.s002.doc]

**S1 Table. The ability of the optimized straining-flotation method to detect and quantify STH eggs in soil.** The egg counts were adjusted for the amount of sample examined. The Ct-value summarized in this table only reflect those that were observed in qPCR-positive samples only.

| **Number of eggs seeded to 100 grams of soil** | **Number of replicates** | **Microscopic examination** | | | | |  | **qPCR** | | | | |
| --- | --- | --- | --- | --- | --- | --- | --- | --- | --- | --- | --- | --- |
|  |  | ***Ascaris*** | |  | ***Trichuris*** | |  | ***Ascaris*** | |  |  | |
|  |  | Sensitivity (%) (95%CI) | Median adjusted egg counts  (minimum; maximum) |  | Sensitivity (%) (95% CI) | Median adjusted egg counts (minimum; maximum) |  | Sensitivity (%) (95%CI) | Median Ct-value  (minimum; maximum) |  | Sensitivity (%) (95%CI) | Median Ct-value  (minimum; maximum) |
| 10 eggs | 8 | 25.0  (0.0; 55.0) | 0.0  (0.0; 2.0) |  | 12.5  (0.0; 35.4) | 0.0  (0.0; 4.0) |  | 12.5  (0.0; 35.4) | 33.5  (33.5; 33.5) |  | 50.0  (15.4; 84.6) | 31.5  (24.4; 35.9) |
| 25 eggs | 8 | 75.0  (45.0; 100) | 4.0  (0.0; 8.0) |  | 62.5  (29.0; 96.0) | 2.0  (0.0; 6.0) |  | 37.5  (4.0; 71.0) | 32.7  (26.3; 34.2) |  | 87.5  (64.6; 100) | 33.3  (25.6; 34.2) |
| 50 eggs | 8 | 100  (100; 100) | 13.0  (8.0; 22.0) |  | 100  (100; 100) | 5.0  (2.0; 10.0) |  | 87.5  (64.6; 100) | 29.6  (24.1; 41.6) |  | 87.5  (64.6; 100) | 29.7  (23.3; 36.9) |
| 100 eggs | 8 | 100  (100; 100) | 36.0  (18.0; 56.0) |  | 100  (100; 100) | 8.0  (6.0; 16.0) |  | 100  (100; 100) | 22.9  (21.1; 37.0) |  | 87.5  (64.6; 100) | 23.4  (22.5; 30.4) |
