# Supplementary material for: Assessment of environmental contamination with soil-transmitted helminths life stages at school compounds, households and open markets in Jimma Town, Ethiopia
Source: PLoS Negl Trop Dis. 2022 Apr 4;16(4):e0010307. doi: 10.1371/journal.pntd.0010307 (PMC9009776; doi:10.1371/journal.pntd.0010307)
Supplement: S2 Table — (DOC) [file pntd.0010307.s003.doc]

**S2 Table. Helminth contamination of soil samples collected in 10 school compounds in Jimma Town, Ethiopia.**

|  |  | | | **N** | | **Any STH** | | | ***Ascaris*** | | | ***Trichuris*** | | | ***Necator*** | | | ***Taenia*** | | | ***Enterobius*** | | | ***Hymenolepis*** | | | ***Strongyloides*** | | | ***Schistosoma*** | | |  |
| --- | --- | --- | --- | --- | --- | --- | --- | --- | --- | --- | --- | --- | --- | --- | --- | --- | --- | --- | --- | --- | --- | --- | --- | --- | --- | --- | --- | --- | --- | --- | --- | --- | --- |
| **School** | | | | | | |  | | |  | | |  | | |  | | |  | | |  | | |  | | |  | | |  | | |
|  | 1 | | 17 | | 88.2 | | | 88.2 | | | 29.4 | | | 0.0 | | | 52.9 | | | 5.9 | | | 5.9 | | | 0.0 | | | 0.0 | | |  | |
|  | 2 | | 17 | | 58.2 | | | 35.3 | | | 41.2 | | | 0.0 | | | 58.8 | | | 11.8 | | | 0.0 | | | 0.0 | | | 0.0 | | |  | |
|  | 3 | | 17 | | 70.6 | | | 64.7 | | | 11.8 | | | 11.8 | | | 41.2 | | | 0.0 | | | 11.8 | | | 0.0 | | | 5.9 | | |  | |
|  | 4 | | 17 | | 76.5 | | | 64.7 | | | 23.5 | | | 0.0 | | | 58.8 | | | 0.0 | | | 5.9 | | | 0.0 | | | 0.0 | | |  | |
|  | 5 | | 17 | | 76.5 | | | 76.5 | | | 17.6 | | | 0.0 | | | 52.9 | | | 0.0 | | | 0.0 | | | 0.0 | | | 0.0 | | |  | |
|  | 6 | | 17 | | 58.8 | | | 58.8 | | | 11.8 | | | 0.0 | | | 52.9 | | | 29.4 | | | 0.0 | | | 0.0 | | | 0.0 | | |  | |
|  | 7 | | 17 | | 52.9 | | | 41.2 | | | 11.8 | | | 0.0 | | | 35.3 | | | 11.8 | | | 0.0 | | | 0.0 | | | 0.0 | | |  | |
|  | 8 | | 17 | | 94.1 | | | 94.1 | | | 5.9 | | | 0.0 | | | 47.1 | | | 17.6 | | | 0.0 | | | 11.8 | | | 0.0 | | |  | |
|  | 9 | | 17 | | 47.1 | | | 47.1 | | | 11.8 | | | 0.0 | | | 58.8 | | | 0.0 | | | 0.0 | | | 0.0 | | | 0.0 | | |  | |
|  | 10 | | 17 | | 41.2 | | | 47.1 | | | 5.9 | | | 0.0 | | | 35.3 | | | 17.6 | | | 5.9 | | | 0.0 | | | 0.0 | | |  | |
| **Location** | | |  | |  | | |  | | |  | | |  | | |  | | |  | | |  | | |  | | |  | | |  | |
|  | Class room | | 60 | | 81.7 | | | 73.3 | | | 26.7 | | | 3.3 | | | 90.0 | | | 26.7 | | | 8.3 | | | 3.3 | | | 0.0 | | |  | |
|  | Play ground | | 60 | | 55.0 | | | 50.0 | | | 11.7 | | | 0.0 | | | 20.0 | | | 0.0 | | | 0.0 | | | 0.0 | | | 0.0 | | |  | |
|  | Latrine | | 50 | | 62.0 | | | 60.0 | | | 12.0 | | | 0.0 | | | 36.0 | | | 0.0 | | | 0.0 | | | 0.0 | | | 2.0 | | |  | |
|  |  | Behind | 10 | | 50.0 | | | 50.0 | | | 0.0 | | | 0.0 | | | 30.0 | | | 0.0 | | | 0.0 | | | 0.0 | | | 0.0 | | |  | |
|  |  | Back yard | 10 | | 50.0 | | | 50.0 | | | 10.0 | | | 0.0 | | | 10.0 | | | 0.0 | | | 0.0 | | | 0.0 | | | 0.0 | | |  | |
|  |  | Entrance girls | 10 | | 70.0 | | | 70.0 | | | 10.0 | | | 0.0 | | | 70.0 | | | 0.0 | | | 0.0 | | | 0.0 | | | 0.0 | | |  | |
|  |  | In front | 10 | | 70.0 | | | 70.0 | | | 0.0 | | | 0.0 | | | 30.0 | | | 0.0 | | | 0.0 | | | 0.0 | | | 0.0 | | |  | |
|  |  | Entrance boys | 10 | | 70.0 | | | 60.0 | | | 40.0 | | | 0.0 | | | 40.0 | | | 0.0 | | | 0.0 | | | 0.0 | | | 10.0 | | |  | |
| **Total** | | | **170** | | **66.5** | | | **61.2** | | | **17.1** | | | **1.2** | | | **49.4** | | | **9.4** | | | **2.9** | | | **1.2** | | | **0.6** | | |  | |
