# Supplementary material for: Assessment of environmental contamination with soil-transmitted helminths life stages at school compounds, households and open markets in Jimma Town, Ethiopia
Source: PLoS Negl Trop Dis. 2022 Apr 4;16(4):e0010307. doi: 10.1371/journal.pntd.0010307 (PMC9009776; doi:10.1371/journal.pntd.0010307)
Supplement: S3 Table — (DOC) [file pntd.0010307.s004.doc]

**S3 Table. Helminth contamination of soil samples collected from 50 households at Jimma Town, Ethiopia.**

|  |  | | **N** | | **Any STH** | | | ***Ascaris*** | | | ***Trichuris*** | | | ***Necator*** | | | ***Taenia*** | | | ***Enterobius*** | | | ***Hymenolepis*** | | | ***Strongyloides*** | | | ***Schistosoma*** | | |  |
| --- | --- | --- | --- | --- | --- | --- | --- | --- | --- | --- | --- | --- | --- | --- | --- | --- | --- | --- | --- | --- | --- | --- | --- | --- | --- | --- | --- | --- | --- | --- | --- | --- |
| **Household** | | | | | |  | | |  | | |  | | |  | | |  | | |  | | |  | | |  | | |  | | |
|  | 1 | 4 | | 75.0 | | | 75.0 | | | 50.0 | | | 0.0 | | | 50.0 | | | 0.0 | | | 0.0 | | | 25.0 | | | 0.0 | | |  | |
|  | 2 | 4 | | 75.0 | | | 75.0 | | | 25.0 | | | 0.0 | | | 25.0 | | | 0.0 | | | 0.0 | | | 0.0 | | | 0.0 | | |  | |
|  | 3 | 4 | | 100.0 | | | 100.0 | | | 0.0 | | | 0.0 | | | 0.0 | | | 0.0 | | | 0.0 | | | 0.0 | | | 0.0 | | |  | |
|  | 4 | 4 | | 100.0 | | | 100.0 | | | 25.0 | | | 0.0 | | | 0.0 | | | 0.0 | | | 0.0 | | | 0.0 | | | 0.0 | | |  | |
|  | 5 | 4 | | 75.0 | | | 75.0 | | | 0.0 | | | 0.0 | | | 25.0 | | | 0.0 | | | 0.0 | | | 0.0 | | | 0.0 | | |  | |
|  | 6 | 4 | | 50.0 | | | 50.0 | | | 25.0 | | | 0.0 | | | 75.0 | | | 0.0 | | | 0.0 | | | 0.0 | | | 0.0 | | |  | |
|  | 7 | 4 | | 100.0 | | | 100.0 | | | 0.0 | | | 0.0 | | | 50.0 | | | 0.0 | | | 0.0 | | | 0.0 | | | 0.0 | | |  | |
|  | 8 | 4 | | 100.0 | | | 100.0 | | | 50.0 | | | 0.0 | | | 50.0 | | | 0.0 | | | 0.0 | | | 0.0 | | | 0.0 | | |  | |
|  | 9 | 4 | | 25.0 | | | 25.0 | | | 25.0 | | | 0.0 | | | 75.0 | | | 25.0 | | | 0.0 | | | 0.0 | | | 0.0 | | |  | |
|  | 10 | 4 | | 25.0 | | | 0.0 | | | 25.0 | | | 0.0 | | | 75.0 | | | 0.0 | | | 0.0 | | | 0.0 | | | 0.0 | | |  | |
|  | 11 | 4 | | 75.0 | | | 75.0 | | | 0.0 | | | 0.0 | | | 50.0 | | | 0.0 | | | 0.0 | | | 0.0 | | | 0.0 | | |  | |
|  | 12 | 4 | | 75.0 | | | 75.0 | | | 75.0 | | | 0.0 | | | 25.0 | | | 0.0 | | | 0.0 | | | 0.0 | | | 0.0 | | |  | |
|  | 13 | 4 | | 100.0 | | | 100.0 | | | 25.0 | | | 0.0 | | | 100.0 | | | 25.0 | | | 0.0 | | | 0.0 | | | 0.0 | | |  | |
|  | 14 | 4 | | 75.0 | | | 75.0 | | | 0.0 | | | 0.0 | | | 75.0 | | | 0.0 | | | 0.0 | | | 0.0 | | | 0.0 | | |  | |
|  | 15 | 4 | | 75.0 | | | 75.0 | | | 25.0 | | | 0.0 | | | 50.0 | | | 0.0 | | | 0.0 | | | 25.0 | | | 0.0 | | |  | |
|  | 16 | 4 | | 75.0 | | | 75.0 | | | 25.0 | | | 0.0 | | | 75.0 | | | 0.0 | | | 0.0 | | | 0.0 | | | 0.0 | | |  | |
|  | 17 | 4 | | 100.0 | | | 100.0 | | | 25.0 | | | 0.0 | | | 75.0 | | | 0.0 | | | 0.0 | | | 0.0 | | | 0.0 | | |  | |
|  | 18 | 4 | | 75.0 | | | 75.0 | | | 0.0 | | | 0.0 | | | 25.0 | | | 0.0 | | | 0.0 | | | 0.0 | | | 25.0 | | |  | |
|  | 19 | 4 | | 25.0 | | | 25.0 | | | 0.0 | | | 0.0 | | | 25.0 | | | 25.0 | | | 0.0 | | | 0.0 | | | 0.0 | | |  | |
|  | 20 | 4 | | 75.0 | | | 75.0 | | | 50.0 | | | 0.0 | | | 100.0 | | | 0.0 | | | 0.0 | | | 0.0 | | | 0.0 | | |  | |
|  | 21 | 4 | | 75.0 | | | 75.0 | | | 0.0 | | | 0.0 | | | 25.0 | | | 0.0 | | | 0.0 | | | 0.0 | | | 0.0 | | |  | |
|  | 22 | 4 | | 75.0 | | | 75.0 | | | 25.0 | | | 0.0 | | | 25.0 | | | 0.0 | | | 0.0 | | | 0.0 | | | 0.0 | | |  | |
|  | 23 | 4 | | 75.0 | | | 50.0 | | | 25.0 | | | 0.0 | | | 50.0 | | | 0.0 | | | 0.0 | | | 0.0 | | | 0.0 | | |  | |
|  | 24 | 4 | | 75.0 | | | 75.0 | | | 25.0 | | | 0.0 | | | 50.0 | | | 0.0 | | | 0.0 | | | 0.0 | | | 0.0 | | |  | |
|  | 25 | 4 | | 75.0 | | | 50.0 | | | 25.0 | | | 0.0 | | | 100.0 | | | 0.0 | | | 0.0 | | | 0.0 | | | 0.0 | | |  | |
|  | 26 | 4 | | 75.0 | | | 75.0 | | | 50.0 | | | 0.0 | | | 50.0 | | | 0.0 | | | 0.0 | | | 0.0 | | | 0.0 | | |  | |
|  | 27 | 4 | | 75.0 | | | 75.0 | | | 0.0 | | | 0.0 | | | 25.0 | | | 0.0 | | | 0.0 | | | 0.0 | | | 0.0 | | |  | |
|  | 28 | 4 | | 50.0 | | | 50.0 | | | 50.0 | | | 0.0 | | | 50.0 | | | 0.0 | | | 25.0 | | | 25.0 | | | 0.0 | | |  | |
|  | 29 | 4 | | 100.0 | | | 100.0 | | | 50.0 | | | 0.0 | | | 100.0 | | | 0.0 | | | 0.0 | | | 25.0 | | | 0.0 | | |  | |
|  | 30 | 4 | | 50.0 | | | 50.0 | | | 0.0 | | | 0.0 | | | 25.0 | | | 0.0 | | | 0.0 | | | 0.0 | | | 0.0 | | |  | |
|  | 31 | 4 | | 100.0 | | | 100.0 | | | 75.0 | | | 0.0 | | | 100.0 | | | 0.0 | | | 25.0 | | | 0.0 | | | 0.0 | | |  | |
|  | 32 | 4 | | 100.0 | | | 100.0 | | | 25.0 | | | 0.0 | | | 50.0 | | | 0.0 | | | 0.0 | | | 0.0 | | | 0.0 | | |  | |
|  | 33 | 4 | | 100.0 | | | 100.0 | | | 0.0 | | | 0.0 | | | 25.0 | | | 0.0 | | | 25.0 | | | 0.0 | | | 0.0 | | |  | |
|  | 34 | 4 | | 100.0 | | | 100.0 | | | 25.0 | | | 0.0 | | | 25.0 | | | 0.0 | | | 0.0 | | | 0.0 | | | 0.0 | | |  | |
|  | 35 | 4 | | 50.0 | | | 50.0 | | | 0.0 | | | 0.0 | | | 50.0 | | | 0.0 | | | 0.0 | | | 0.0 | | | 0.0 | | |  | |
|  | 36 | 4 | | 100.0 | | | 100.0 | | | 50.0 | | | 0.0 | | | 25.0 | | | 0.0 | | | 0.0 | | | 0.0 | | | 0.0 | | |  | |
|  | 37 | 4 | | 100.0 | | | 100.0 | | | 0.0 | | | 0.0 | | | 75.0 | | | 0.0 | | | 0.0 | | | 0.0 | | | 0.0 | | |  | |
|  | 38 | 4 | | 25.0 | | | 25.0 | | | 0.0 | | | 0.0 | | | 50.0 | | | 0.0 | | | 0.0 | | | 0.0 | | | 0.0 | | |  | |
|  | 39 | 4 | | 75.0 | | | 75.0 | | | 0.0 | | | 0.0 | | | 100.0 | | | 0.0 | | | 0.0 | | | 0.0 | | | 0.0 | | |  | |
|  | 40 | 4 | | 75.0 | | | 75.0 | | | 0.0 | | | 0.0 | | | 50.0 | | | 0.0 | | | 0.0 | | | 0.0 | | | 0.0 | | |  | |
|  | 41 | 4 | | 75.0 | | | 75.0 | | | 25.0 | | | 0.0 | | | 75.0 | | | 0.0 | | | 0.0 | | | 0.0 | | | 0.0 | | |  | |
|  | 42 | 4 | | 25.0 | | | 25.0 | | | 0.0 | | | 0.0 | | | 25.0 | | | 0.0 | | | 0.0 | | | 0.0 | | | 0.0 | | |  | |
|  | 43 | 4 | | 50.0 | | | 50.0 | | | 25.0 | | | 0.0 | | | 75.0 | | | 0.0 | | | 0.0 | | | 0.0 | | | 0.0 | | |  | |
|  | 44 | 4 | | 75.0 | | | 75.0 | | | 25.0 | | | 0.0 | | | 75.0 | | | 0.0 | | | 0.0 | | | 0.0 | | | 0.0 | | |  | |
|  | 45 | 4 | | 100.0 | | | 75.0 | | | 100.0 | | | 0.0 | | | 25.0 | | | 0.0 | | | 0.0 | | | 0.0 | | | 0.0 | | |  | |
|  | 46 | 4 | | 75.0 | | | 50.0 | | | 0.0 | | | 25.0 | | | 75.0 | | | 0.0 | | | 0.0 | | | 0.0 | | | 0.0 | | |  | |
|  | 47 | 4 | | 75.0 | | | 75.0 | | | 0.0 | | | 0.0 | | | 0.0 | | | 0.0 | | | 0.0 | | | 25.0 | | | 0.0 | | |  | |
|  | 48 | 4 | | 100.0 | | | 100.0 | | | 100.0 | | | 0.0 | | | 50.0 | | | 0.0 | | | 0.0 | | | 0.0 | | | 0.0 | | |  | |
|  | 49 | 3 | | 33.3 | | | 33.3 | | | 33.3 | | | 0.0 | | | 66.7 | | | 0.0 | | | 0.0 | | | 0.0 | | | 0.0 | | |  | |
|  | 50 | 4 | | 50.0 | | | 50.0 | | | 0.0 | | | 0.0 | | | 25.0 | | | 0.0 | | | 0.0 | | | 0.0 | | | 0.0 | | |  | |
| **Location** | |  | |  | | |  | | |  | | |  | | |  | | |  | | |  | | |  | | |  | | |  | |
|  | Back yard | 50 | | 74.0 | | | 72.0 | | | 26.0 | | | 0.0 | | | 48.0 | | | 2.0 | | | 2.0 | | | 2.0 | | | 0.0 | | |  | |
|  | House entrance | 50 | | 72.0 | | | 72.0 | | | 20.0 | | | 0.0 | | | 50.0 | | | 2.0 | | | 2.0 | | | 0.0 | | | 0.0 | | |  | |
|  | Kitchen | 49 | | 65.3 | | | 59.2 | | | 18.4 | | | 2.0 | | | 53.1 | | | 4.1 | | | 2.0 | | | 6.0 | | | 2.0 | | |  | |
|  | Latrine entrance | 50 | | 84.0 | | | 82.0 | | | 30.0 | | | 0.0 | | | 54.0 | | | 0.0 | | | 0.0 | | | 2.0 | | | 2.0 | | |  | |
| **Total** | | **199** | | **73.9** | | | **71.4** | | | **23.6** | | | **0.5** | | | **51.3** | | | **2.0** | | | **1.5** | | | **2.5** | | | **0.5** | | |  | |
