# Supplementary material for: Assessment of environmental contamination with soil-transmitted helminths life stages at school compounds, households and open markets in Jimma Town, Ethiopia
Source: PLoS Negl Trop Dis. 2022 Apr 4;16(4):e0010307. doi: 10.1371/journal.pntd.0010307 (PMC9009776; doi:10.1371/journal.pntd.0010307)
Supplement: S4 Table — (DOC) [file pntd.0010307.s005.doc]

**S4 Table. Helminth contamination of soil samples collected from 9 markets at Jimma Town, Ethiopia.**

|  | |  | **N** | **Any STH** | ***Ascaris*** | ***Trichuris*** | ***Necator*** | ***Taenia*** | ***Enterobius*** | ***Hymenolepis*** | ***Strongyloides*** | ***Schistosoma*** |
| --- | --- | --- | --- | --- | --- | --- | --- | --- | --- | --- | --- | --- |
| **Market** | | | |  |  |  |  |  |  |  |  |  |
|  | | 1 | 3 | 66.7 | 33.3 | 66.7 | 0.0 | 33.3 | 0.0 | 33.3 | 0.0 | 0.0 |
|  | | 2 | 3 | 0.0 | 0.0 | 0.0 | 0.0 | 66.7 | 33.3 | 0.0 | 0.0 | 0.0 |
|  | | 3 | 6 | 33.3 | 33.3 | 0.0 | 0.0 | 83.3 | 16.7 | 0.0 | 0.0 | 0.0 |
|  | | 4 | 3 | 100.0 | 100.0 | 0.0 | 0.0 | 100.0 | 0.0 | 0.0 | 0.0 | 0.0 |
|  | | 5 | 3 | 100.0 | 100.0 | 66.7 | 0.0 | 100.0 | 33.3 | 0.0 | 0.0 | 0.0 |
|  | | 6 | 3 | 100.0 | 100.0 | 66.7 | 0.0 | 100.0 | 0.0 | 0.0 | 0.0 | 0.0 |
|  | | 7 | 3 | 100.0 | 0.0 | 66.7 | 0.0 | 66.7 | 0.0 | 0.0 | 0.0 | 0.0 |
|  | | 8 | 3 | 33.3 | 0.0 | 33.3 | 0.0 | 66.7 | 0.0 | 0.0 | 0.0 | 0.0 |
|  | | 9 | 3 | 100.0 | 0.0 | 33.3 | 0.0 | 66.7 | 0.0 | 0.0 | 0.0 | 0.0 |
| **Location** | | |  |  |  |  |  |  |  |  |  |  |
|  | Fruit | | 10 | 60.0 | 50.0 | 40.0 | 0.0 | 80.0 | 0.0 | 0.0 | 0.0 | 0.0 |
|  | Grains | | 10 | 70.0 | 40.0 | 50.0 | 0.0 | 80.0 | 10.0 | 10.0 | 0.0 | 0.0 |
|  | Vegetables | | 10 | 70.0 | 70.0 | 10.0 | 0.0 | 70.0 | 20.0 | 0.0 | 0.0 | 0.0 |
| **Total** | | | **30** | **66.7** | **53.3** | **33.3** | **0.0** | **76.7** | **10.0** | **3.3** | **0.0** | **0.0** |
