# Supplementary material for: Assessment of environmental contamination with soil-transmitted helminths life stages at school compounds, households and open markets in Jimma Town, Ethiopia
Source: PLoS Negl Trop Dis. 2022 Apr 4;16(4):e0010307. doi: 10.1371/journal.pntd.0010307 (PMC9009776; doi:10.1371/journal.pntd.0010307)
Supplement: S5 Table — (DOC) [file pntd.0010307.s006.doc]

**S5 Table. STH infections in 10 schools in Jimma Town, Ethiopia.**

| **School** | **N** | **Prevalence any STH (%)** | ***Ascaris*** |  |  | ***Trichuris*** |  |  | **Hookworm** |  |
| --- | --- | --- | --- | --- | --- | --- | --- | --- | --- | --- |
|  |  |  | Prevalence (%) | Mean fecal egg counts (EPG) |  | Prevalence (%) | Mean fecal egg counts (EPG) |  | Prevalence (%) | Mean fecal egg counts (EPG) |
| 1 | 60 | 31.7 | 11.7 | 290 |  | 20 | 17.6 |  | 11.7 | 22.4 |
| 2 | 60 | 26.7 | 21.7 | 2483.2 |  | 20 | 26.8 |  | 0 | 0 |
| 3 | 60 | 26.7 | 6.7 | 365.2 |  | 25 | 83.2 |  | 0 | 0 |
| 4 | 60 | 31.7 | 10 | 1236.8 |  | 23.3 | 32.4 |  | 3.3 | 1.2 |
| 5 | 60 | 55 | 28.3 | 4550.8 |  | 38.3 | 162.8 |  | 5 | 10 |
| 6 | 60 | 8.3 | 3.3 | 2.8 |  | 3.3 | 8 |  | 3.3 | 2.8 |
| 7 | 60 | 23.3 | 15 | 928.8 |  | 8.3 | 14.4 |  | 5 | 8.8 |
| 8 | 60 | 26.7 | 18.3 | 1389.6 |  | 16.7 | 77.2 |  | 0 | 0 |
| 9 | 60 | 26.7 | 6.7 | 3509.6 |  | 11.7 | 50.4 |  | 16.7 | 45.6 |
| 10 | 60 | 23.3 | 11.7 | 380.8 |  | 15 | 62.4 |  | 5 | 5.2 |
