# Supplementary material for: Assessment of environmental contamination with soil-transmitted helminths life stages at school compounds, households and open markets in Jimma Town, Ethiopia
Source: PLoS Negl Trop Dis. 2022 Apr 4;16(4):e0010307. doi: 10.1371/journal.pntd.0010307 (PMC9009776; doi:10.1371/journal.pntd.0010307)
Supplement: S1 Info — (DOC) [file pntd.0010307.s007.doc]

**S1 Info. The questionnaire to gain insights into the characteristics of the households.**

| **Q1.** | Study area |  |
| --- | --- | --- |
| **Q2.** | Number of household members |  |
| **Q3.** | Age of youngest child (in months) |  |
| **Q4.** | Presence of animals in compound | Yes/No |
| **Q5.** | Presence of latrine | Yes/No |
| **Q6.** | Presence of walls around latrine | Yes/No |
| **Q7.** | Presence of latrine door | Yes/No |
| **Q8.** | Presence of latrine roof | Yes/No |
| **Q9.** | Flies observed in/around defecation area | Yes/No |
| **Q10.** | Visible stool observed on household areas | Yes/No |
| **Q12.** | Does any member of your family defecate outside of latrine? | Yes/No |
| **Q13.** | Presence of water in household compound | Yes/No |
| **Q14.** | Type of kitchen | Open space; Soil floor with wall and roof; Cement floor with wall and roof |
| **Q15.** | Distance from the household entrance to the latrine entrance in meter |  |
