# Supplementary material for: Assessment of environmental contamination with soil-transmitted helminths life stages at school compounds, households and open markets in Jimma Town, Ethiopia
Source: PLoS Negl Trop Dis. 2022 Apr 4;16(4):e0010307. doi: 10.1371/journal.pntd.0010307 (PMC9009776; doi:10.1371/journal.pntd.0010307)
Supplement: S2 Info — (DOC) [file pntd.0010307.s008.doc]

**S2 Info. The questionnaire on knowledge, attitude, and practice towards soil-transmitted helminths**

| School Name | | |  |
| --- | --- | --- | --- |
| Study participant ID | | |  |
| Q1 What is your age? (in years) | | | |  |  | | --- | --- | |
| Q2 What is the sex of the child?  (M= male; F = Female) | | | |  | | --- | |
| Q3 Which religions do you practice? | | | |
|  | Christian | | |  | | --- | |
|  | Muslim | | |  | | --- | |
|  | Other | | _________________________ |
| Q4 What is the occupation of your father? | | | |
|  | Farmer | | |  | | --- | |
|  | Government employee | | |  | | --- | |
|  | Private employee | | |  | | --- | |
|  | Other | | ________________________ |
| Q5 What is your father’s educational status? | | | |
|  | Illiterate | | |  | | --- | |
|  | Elementary school | | |  | | --- | |
|  | Junior high school | | |  | | --- | |
|  | Senior high school | | |  | | --- | |
|  | Diploma/university | | |  | | --- | |
|  | I do not know this | | |  | | --- | |
| Q6 What is the occupation of your mother? | | | |
|  | Farmer | | |  | | --- | |
|  | Government employee | | |  | | --- | |
|  | Private employee | | |  | | --- | |
|  | Other | | ________________________ |
| Q7 What is your mother’s educational status | | | |
|  | Illiterate | | |  | | --- | |
|  | Elementary school | | |  | | --- | |
|  | Junior high school | | |  | | --- | |
|  | Senior high school | | |  | | --- | |
|  | Diploma/university | | |  | | --- | |
|  | I do not know this | | |  | | --- | |
| Q8 Which names have you heard before? Multiple answers possible. | | | |
|  | *Ascaris* | | |  | | --- | |
|  | HIV/Aids | | \|  \| \| --- \| |
|  | Hookworms | | \|  \| \| --- \| |
|  | Intestinal worms | | \|  \| \| --- \| |
|  | Schistosomiasis | | \|  \| \| --- \| |
|  | *Trichuris* | | \|  \| \| --- \| |
|  | Tuberculosis | |  |
|  | Malaria | | \|  \| \| --- \| |
|  | I have never heard these names | | \|  \| \| --- \| |
| Q9 Who told you about these names? Multiple answers possible. | | | |
|  | Health extension worker | |  | | --- | | |
|  | My family (parents, brothers/sisters) | |  | | --- | | |
|  | My teacher at school | |  | | --- | | |
|  | Nurse / doctor at health Centre | |  | | --- | | |
|  | Presenter at the radio | |  | | --- | | |
|  | Presenter at the television | |  | | --- | | |
|  | Some-one else | |  | | --- | | |
|  | I cannot remember who first told me | |  | | --- | | |
| Q10 What do(es) Ascaris/Trichuris/hookworm/intestinal worms with your body? Multiple answers possible. | | | |
|  | They/it suck(s) my blood | |  | | --- | | |
|  | They/it eat(s) my food | |  | | --- | | |
|  | They/it give(s) me stomachache | |  | | --- | | |
|  | They/it make(s) me blind | |  | | --- | | |
|  | They/it causes bubbles on my skin | |  | | --- | | |
|  | They/it makes me urinate blood | |  | | --- | | |
|  | I do not know what they/it do(es) | |  | | --- | | |
| Q11 Which part of your body does Ascaris/Trichuris/hookworm/intestinal worms live? Multiple answers possible. | | | |
|  | My eyes | |  | | --- | | |
|  | My head | |  | | --- | | |
|  | My heart |  | |
|  | My intestine | |  | | --- | | |
|  | My lungs | |  | | --- | | |
|  | My skin | |  | | --- | | |
|  | I do not know where they/it live(s) | |  | | --- | | |
| Q12 How do (es) Ascaris/Trichuris/hookworm/intestinal worms spread among people? Multiple answers possible. | | | |
|  | Through air | |  | | --- | | |
|  | Through bites of insects (e.g. mosquitos) | |  | | --- | | |
|  | Through blood | |  | | --- | | |
|  | Through eating of raw meat | |  | | --- | | |
|  | Through saliva | |  | | --- | | |
|  | Through stool | |  | | --- | | |
|  | None of the above | |  | | --- | | |
|  | I do not know how they/it spread(s) | |  | | --- | | |
| Q13. How many children in your school have Ascaris/Trichuris/hookworm/intestinal worms | | | |
|  | No one has worms | |  | | --- | | |
|  | Less than half of the school | |  | | --- | | |
|  | More than half of the school | |  | | --- | | |
|  | I do not know | |  | | --- | | |
| Q14. What can doctors do against Ascaris/Trichuris/hookworm/intestinal worms . Multiple answers possible. | | | |
|  | Give a medicine | |  | | --- | | |
|  | Give a vaccination | |  | | --- | | |
|  | There is nothing they can do | |  | | --- | | |
|  | I do not know what they can do | |  | | --- | | |
| Q15 Where do you usually defecate? | | | |
|  | in the latrine/toilet of my house | |  | | --- | | |
|  | in the latrine/toilet in the compound | |  | | --- | | |
|  | in a latrine/toilet of my school | |  | | --- | | |
|  | around/outside the compound/ school (in bush) | |  | | --- | | |
|  | I don’t know / I refuse to tell you | |  | | --- | | |
| Q16 Where did you defecate last time? | | | |
|  | in the latrine/toilet of my house | |  | | --- | | |
|  | in the latrine/toilet in the compound | |  | | --- | | |
|  | in a latrine/toilet of my school | |  | | --- | | |
|  | around/outside the compound/ school (in bush) | |  | | --- | | |
|  | I don’t know / I refuse to tell you | |  | | --- | | |
| Q17 Do you wash your hands before eating? | | | |
|  | Always | |  | | --- | | |
|  | Sometimes | |  | | --- | | |
|  | Never | |  | | --- | | |
|  | I don’t know / I refuse to tell you | |  | | --- | | |
| Q18 Do you wash your hands after using toilet? | | | |
|  | Always | |  | | --- | | |
|  | Sometimes | |  | | --- | | |
|  | Never | |  | | --- | | |
|  | I don’t know / I refuse to tell you | |  | | --- | | |
| Q19 How do you wash your hands? | | | |
|  | With only water | |  | | --- | | |
|  | With water and soap | |  | | --- | | |
|  | With water and ash | |  | | --- | | |
|  | Other | ____________________________ | |
|  | I don’t wash my hands / I refuse to tell you | |  | | --- | | |
| Q20 Are fingers trimmed | | | |
|  | Yes | |  | | --- | | |
|  | No | |  | | --- | | |
| Q21 If you say yes for Q20 how often do you trim your nails? | | | |
|  | One time per week | |  | | --- | | |
|  | One time per two weeks | |  | | --- | | |
|  | Less than one time per month | |  | | --- | | |
|  | I don’t know / I refuse to tell you | |  | | --- | | |
| Q22 Do you wear shoe when you go out of your home? | | | |
|  | Yes | |  | | --- | | |
|  | No | |  | | --- | | |
| Q23 If you say yes Q22 how often do you wear your shoes? | | | |
|  | Always | |  | | --- | | |
|  | Sometimes | |  | | --- | | |
|  | Seldom | |  | | --- | | |
| Q24 Do you think that you are at risk of getting worms (STHs) infection from household or school compound? | | | |
|  | Yes | |  | | --- | | |
|  | NO | |  | | --- | | |
|  | I don’t know / I refuse to tell you | |  | | --- | | |
| Q25 Would you be anxious if you got worm infection? | | | |
|  | Yes |  | |
|  | No |  | |
|  | Do not know/refuse to tell |  | |
